# CdrS Is a Global Transcriptional Regulator Influencing Cell Division in Haloferax volcanii

**DOI:** 10.1128/mBio.01416-21

**Published:** 2021-07-13

**Authors:** Yan Liao, Verena Vogel, Sabine Hauber, Jürgen Bartel, Omer S. Alkhnbashi, Sandra Maaß, Thandi S. Schwarz, Rolf Backofen, Dörte Becher, Iain G. Duggin, Anita Marchfelder

**Affiliations:** a The ithree institute, University of Technology Sydney, Ultimo, Australia; b Biology II, Ulm University, Ulm, Germany; c Department of Microbial Proteomics, Institute of Microbiology, University of Greifswald, Greifswald, Germany; d Bioinformatics Group, Department of Computer Science, University of Freiburg, Freiburg, Germany; e Signalling Research Centres BIOSS and CIBSS, University of Freiburg, Freiburg, Germany; University of Vienna

**Keywords:** *Archaea*, *Haloferax volcanii*, cell division, small protein, transcriptional regulation

## Abstract

Transcriptional regulators that integrate cellular and environmental signals to control cell division are well known in bacteria and eukaryotes, but their existence is poorly understood in archaea. We identified a conserved gene (*cdrS*) that encodes a small protein and is highly transcribed in the model archaeon Haloferax volcanii. The *cdrS* gene could not be deleted, but CRISPR interference (CRISPRi)-mediated repression of the *cdrS* gene caused slow growth and cell division defects and changed the expression of multiple genes and their products associated with cell division, protein degradation, and metabolism. Consistent with this complex regulatory network, overexpression of *cdrS* inhibited cell division, whereas overexpression of the operon encoding both CdrS and a tubulin-like cell division protein (FtsZ2) stimulated division. Chromatin immunoprecipitation-DNA sequencing (ChIP-Seq) identified 18 DNA-binding sites of the CdrS protein, including one upstream of the promoter for a cell division gene, *ftsZ1*, and another upstream of the essential gene *dacZ*, encoding diadenylate cyclase involved in c-di-AMP signaling, which is implicated in the regulation of cell division. These findings suggest that CdrS is a transcription factor that plays a central role in a regulatory network coordinating metabolism and cell division.

## INTRODUCTION

Cell division is a central aspect of the biology of all living organisms. In almost all bacteria, cell division is mediated by a ring-like division complex, or divisome, assembled around FtsZ, the ancestral homolog of eukaryotic tubulins that form the network of microtubules as part of the cytoskeleton (1). Bacterial FtsZ polymerizes into dynamic filaments and then assembles the contractile “Z-ring” structure around the middle plane of the cell to constrict during cell division (2). FtsZ is thought to perform multiple functions, including recruiting divisome proteins to the division site (2), effecting membrane constriction (3), and guiding cell wall synthesis (4, 5). It is well known that bacteria can tightly coordinate division with growth rate to accurately duplicate their genomes and to homeostatically regulate their cell sizes (6, 7). A number of metabolic enzymes/pathways have been shown to directly regulate division in response to nutrient/metabolic status, by modulating the activity and assembly of FtsZ to ensure faithful cell division (8–10). Bacteria also regulate cell division in response to stresses including DNA damage. In Escherichia coli, the DNA damage response, or SOS response, induces the expression of many genes, including FtsZ-specific inhibitors that block division. After the SOS response is turned off, the cell division inhibitor is degraded by proteases, allowing cell division to resume (11).

Almost all bacterial species contain only one FtsZ (12), whereas many archaea carry two distinct FtsZs (FtsZ1 and FtsZ2) (13, 14). Haloferax volcanii has been proposed as a powerful model for understanding archaeal cell division and morphology (14–16). A recent study used H. volcanii to identify a new mechanism of FtsZ-based cell division: FtsZ1 has an initial scaffold-like function to stabilize the machinery controlling cell division and shape, and FtsZ2 is more actively involved in division constriction (16). However, how archaea regulate cell division in response to their environment and metabolism is not understood. A recent study has shown that metal micronutrients in the growth medium affect the cell size and shape of H. volcanii, suggesting a potential link between nutrient availability and the regulation of cell division (17). Another regulator of cell division in archaea may be a second messenger, cyclic di-AMP (c‐di‐AMP), which was shown to be essential and tightly regulated in H. volcanii (18). Alteration of c-di-AMP levels in H. volcanii changed the average cell size in a low-salt medium, implying a function of c-di-AMP in the regulation of cell size and division.

**FIG 1:**
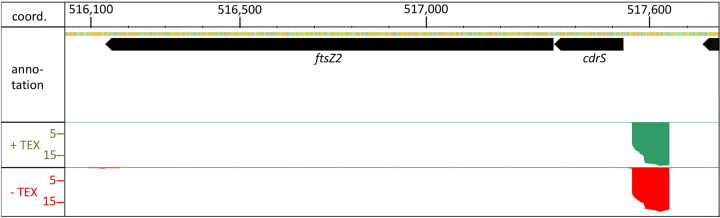
Genomic location of the *cdrS* gene. The gene for the small protein CdrS is upstream from the *ftsZ2* gene, which encodes a homolog of the bacterial cell division protein FtsZ. The genes are separated by only two nucleotides. Differential RNA-Seq (dRNA-Seq) data (38) (bottom panels) reveal a strong promoter upstream from the *cdrS* gene (data were visualized with the Integrated Genome Browser [73]). Red signals (−TEX) represent reads from an RNA fraction containing all cellular RNAs (primary transcripts as well as processed transcripts, read depth of ×10^3^). Green signals (+TEX) represent reads from an RNA sample treated with terminator 5′ phosphate-dependent exonuclease (TEX) (read depth of ×10^3^), resulting in enrichment of primary transcripts. Comparison of both data sets allows determination of transcription start sites. The genome coordinates and the annotation are shown at the top.

The current annotation of the H. volcanii genome shows 4,105 annotated protein-encoding genes (HaloLex 26.11.19) (19), and 316 of these open reading frames (ORFs) code for small proteins of 70 amino acids (we define small proteins here as proteins of 70 or less amino acids) or fewer (20). Recent data show that proteins smaller than 70 amino acids are common and fulfil important functions in members of the *Bacteria* and *Eukarya* (reviewed in references 21–27). Until more recently, sequences encoding such small proteins had long been overlooked and omitted from functional analyses (22, 28). Small proteins of *Archaea* have only been addressed in a few studies, which have implicated them in the regulation of nitrogen metabolism, protein degradation, oxidative stress response, and sulfur metabolism (29–37). This limited body of information suggests a great potential of small proteins in the regulation of archaeal metabolism and biology. Quantitative proteome analysis of H. volcanii under standard conditions and two stress conditions identified 60 of the annotated 316 small proteins predicted in H. volcanii (20). We have identified a small protein-encoding gene (HVO_0582) adjacent to *ftsZ2* in H. volcanii that is highly transcribed according to a transcription start site analysis (Fig. 1) (38). Based on the results reported here and in accordance with the concurrent study of its homologue from Halobacterium salinarum (39), we termed this protein CdrS (cell division regulator, short).

The *cdrS-ftsZ2* locus is well conserved across the *Euryarchaeota*, especially within the class of *Halobacteria* (39). Using H. volcanii as the model organism, we found that the *cdrS* gene is essential, and unlike *ftsZ2*, could not be deleted. As *cdrS* encodes a predicted transcription regulator, we used an integrative approach to investigate its functions by combining gene repression by CRISPR interference (CRISPRi), chromatin immunoprecipitation-DNA sequencing (ChIP-Seq), transcriptomics, quantitative proteomics, and microscopy. Our data suggest that CdrS in *H. volcanii* is a global transcriptional regulator, controlling *ftsZ* expression and genes linked to other metabolic and regulatory processes. This may allow cells to properly coordinate growth, division, and metabolic activity.

## RESULTS

### Repression of *cdrS-ftsZ2* expression causes cell growth defects in H. volcanii.

CdrS is predicted to be a small protein of 61 amino acids (we define small proteins here as proteins of ≤70 amino acids) with an isoelectric point of 9.5, which is very basic for a halophilic protein and might indicate that it binds to negatively charged molecules like nucleic acids. The *cdrS* open reading frame (ORF) is located three nucleotides upstream from the *ftsZ2* ORF, suggesting that they might be transcribed together (Fig. 1). The currently predicted function of CdrS is a transcriptional regulator containing the CopG/Arc/MetJ DNA-binding domain with a ribbon-helix-helix (RHH) motif. To our knowledge, similar small transcription factors have so far only been described for bacteria (40, 41).

**FIG 2:**
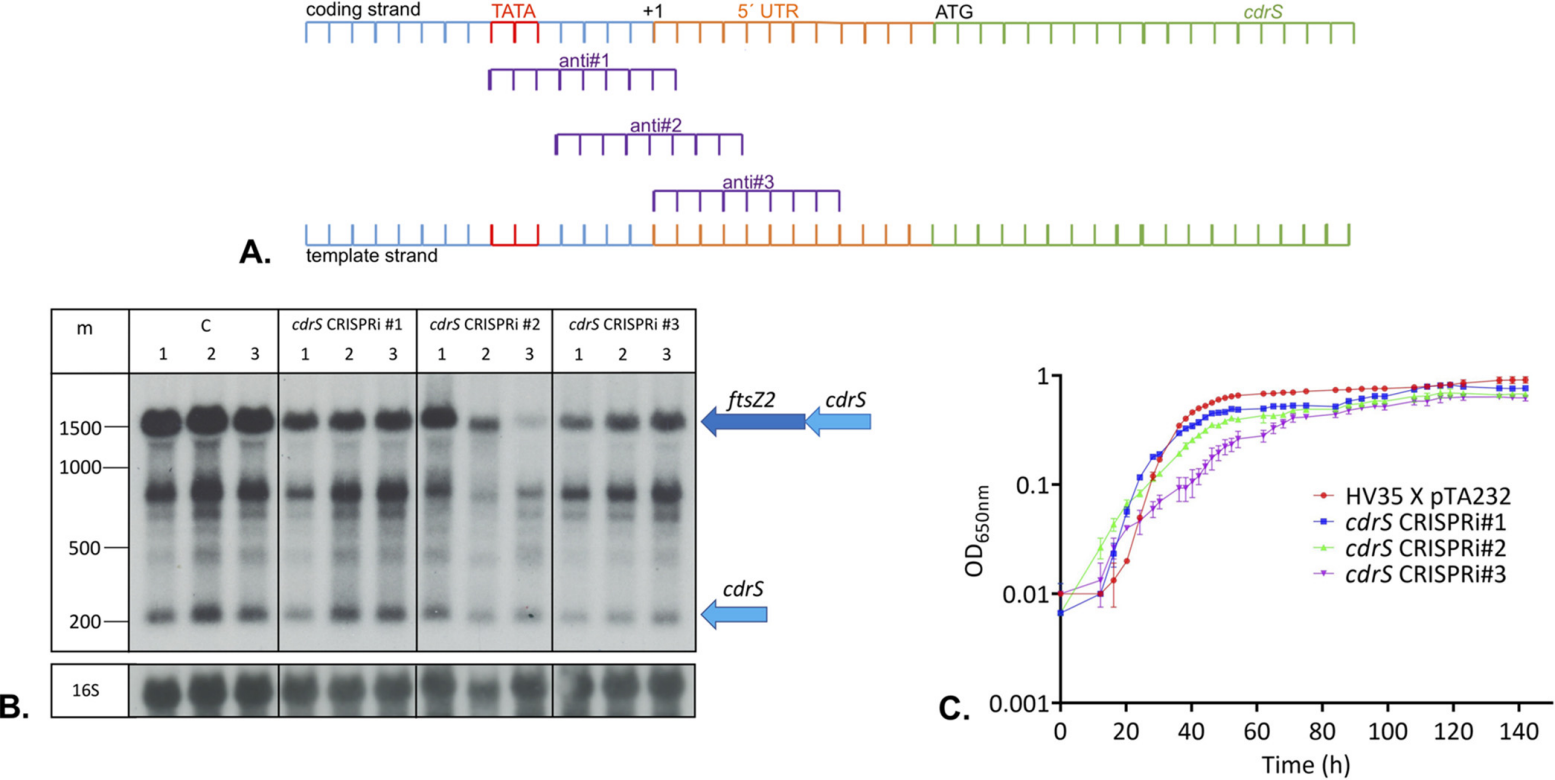
Repression of *cdrS-ftsZ2* and its effect on growth. (A) Locations of crRNAs directed against the *cdrS* gene. Both strands of the *cdrS* upstream region are shown. Three different crRNAs (anti#1, -#2, and -#3) were designed to target the template strand in the promoter region close to the position corresponding to the transcription start site. The TATA box is shown in red, the transcription start site is indicated as “+1,” the 5′ untranslated region (UTR) is shown in orange, the open reading frame is in green, and the ATG start codon is indicated. (B) Both *cdrS* and *ftsZ2* are repressed by CRISPRi. Hybridization with a probe against the *cdrS* mRNA (top panel) revealed the monocistronic *cdrS* mRNA (signal at about 250 nucleotides), as well as the bicistronic *cdrS*-*ftsZ2* mRNA (signal at about 1,500 nucleotides) and intermediate RNAs that may be degradation products. Lanes under C, wild-type RNA from HV30 strain with pTA232 plasmid without insert (HV30 × pTA232); lanes under *cdrS* CRISPRi #1, #2, and #3, HV30 strain expressing crRNAs anti#1, anti#2, or anti#3 (HV30 × pTA232-tele-0582anti#1, -#2, and -#3). Experiments were done in biological triplicates (lanes 1, 2, and 3 in each case). Bottom, hybridization with a probe against the 16S rRNA. An RNA size marker (m) is given at the left in nucleotides. The mono- and bicistronic mRNAs are shown at the right schematically. (C) Growth of the *cdrS* CRISPRi strains is impaired. Growth of wild-type *Haloferax* strain (HV35 × pTA232) was compared to growth of the CRISPRi strains expressing crRNA anti#1, crRNA anti#2, or crRNA anti#3 (*cdrS* CRISPR#1, -#2, and -#3). Analyses were done in triplicate; standard deviations are shown as error bars. *x* axis shows the time of growth, and *y* axis shows the OD semilogarithmically.

To help uncover the biological functions of CdrS, we first attempted to generate a *cdrS* deletion mutant to observe its functional consequences. Several attempts to generate such a strain using the standard pop-in/pop-out method (42) proved unsuccessful, suggesting that *cdrS* is essential. Therefore, we employed CRISPRi to repress the expression of *cdrS* (Fig. S1A in the supplemental material). The CRISPRi approach takes advantage of the endogenous CRISPR-Cas system of H. volcanii that can be harnessed to repress transcription. It was already successfully used in H. volcanii to repress the expression of several genes (43, 44). Three different CRISPR RNAs (crRNAs) were designed that bind to the promoter and transcription start site regions of *cdrS* and guide the endogenous Cascade complex (complex of Cas proteins Cas5, -6b, -7, and -8b) to these sequences, thereby preventing transcription initiation (Fig. 2A). H. volcanii cells were transformed with plasmids for expression of the three crRNAs, and Northern blot analyses showed that, in wild-type cells (strains HV30 and HV35; the HV30 and HV35 strains that express the crRNAs are termed the CRISPRi strains herein), the bicistronic *cdrS*-*ftsZ2* mRNA was the predominant transcript (∼1,500 nucleotides [nt]), and that crRNAs anti#1, anti#2, and anti#3 repressed its expression moderately, to averages of 93%, 76%, and 60% of the wild-type expression levels, respectively (Fig. 2B; Fig. S1B). The monocistronic *cdrS* mRNA (∼250 nt) was repressed by all three crRNAs (57%, 42%, and 40% of the wild-type, respectively) (Fig. 2B; Fig. S1B). Additional RNAs were also observed, which could be generated by cleavage of the longer bicistronic mRNA or by premature termination of transcription. All three CRISPRi strains showed reduced growth rates in comparison to the control strain expressing no crRNA (Fig. 2C), with doubling times of 3.9 ± 0.15 h (mean ± standard deviation) for the wild-type control (HV35 × pTA232), 4.2 ± 0.07 h for the *cdrS* CRISPRi#1 strain, 8.4 ± 0.4 h for the *cdrS* CRISPRi#2 strain, and 8.9 ± 0.84 h for the *cdrS* CRISPRi#3 strain.

10.1128/mBio.01416-21.1 FIG S1 (A.A) CRISPRi inhibits transcription initiation. The mini-CRISPRi plasmid used for expression of crRNAs. The plasmid contains the CRISPR leader sequence, which contains the promoter, one crRNA spacer flanked by two repeats, and a synthetic terminator. The crRNA is expressed from the leader promoter and terminated by a synthetic terminator. The *leuB* gene is used as a marker gene for selection in H. volcanii. (A.B) Schematic illustration of CRISPRi targeting of the *cdrS* promoter region in H. volcanii. The Cascade complex (complex of Cas proteins Cas 5, 6b, 7, and 8b) binds crRNA anti#1, anti#2, or anti#3 expressed from the plasmid (A.A) to form the Cascade/crRNA complex. The Cascade/crRNA complex is guided by the crRNA to bind to the target DNA sequence at the promoter and TSS region of *cdrS*, thereby preventing transcription initiation. (B.A and B.B) Quantification of *cdrS* and *cdrS-ftsZ2* repression. Signal intensity of the bicistronic *cdrS*-*ftsZ2* mRNA (B.A) and the *cdrS* mRNA (B.B) was measured with ImageJ and set into relation to the 16S rRNA signal that was used as loading control. The proportion of the detected RNA is given as the percentage compared to cells carrying a control plasmid. The amount of RNA in the control strains was set to 100% (bars labeled C), and the amounts of RNA in strains expressing a targeting spacer (the three CRISPRi strains expressing anti#1, -#2, and -#3) were set relative to the control. The strongest repression of the *cdrS* mRNA was obtained upon expression of crRNA anti#3. Download FIG S1, PDF file, 0.3 MB. Copyright © 2021 Liao et al. 2021 Liao et al. https://creativecommons.org/licenses/by/4.0/ This content is distributed under the terms of the Creative Commons Attribution 4.0 International license.

### Repression of *cdrS-ftsZ2* causes changes in the transcriptome.

To determine whether *cdrS-ftsZ2* repression influences the expression of other genes, we compared the transcriptomes of the CRISPRi strain *cdrS* CRISPRi#2 and the wild-type strain (Tables S1A and S4A).

**FIG 3:**
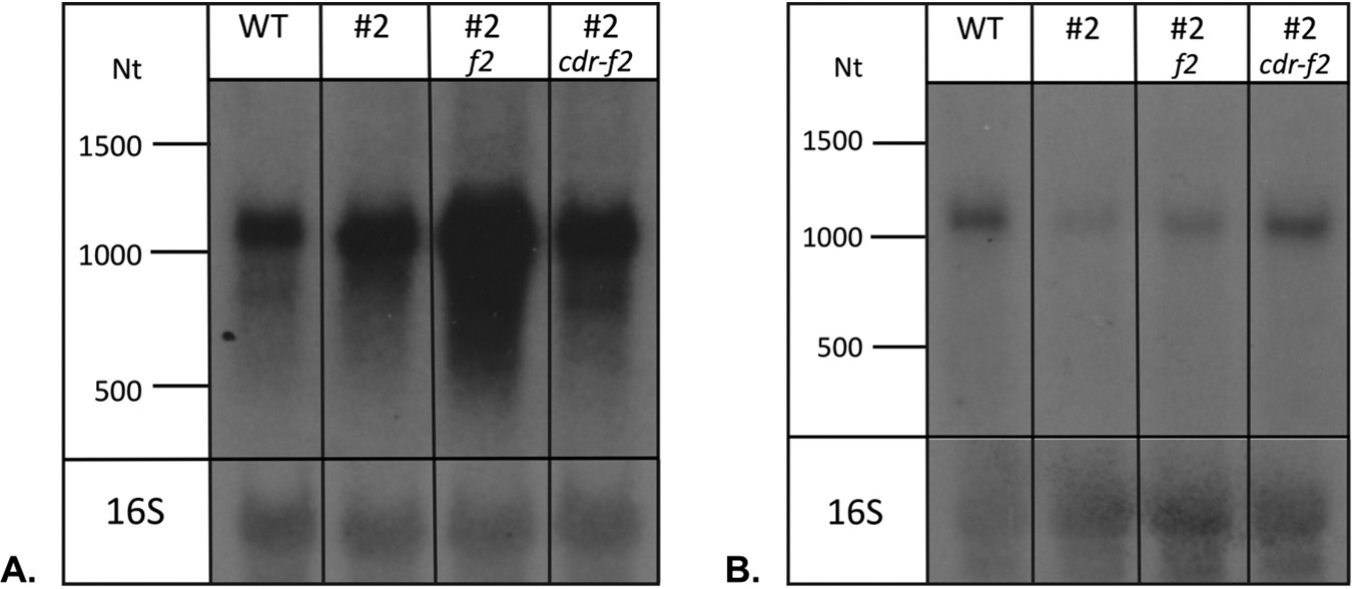
Northern analyses reveal specific effects of repression of *cdrS* only. RNA was analyzed from wild-type cells (lane WT) (HV30 × pTA232 × pTA409), *cdrS* CRISPRi#2 cells (lane #2) (HV30 × pTA232-tele-0582anti#2 × pTA409), and *cdrS* CRISPRi#2 cells complemented with *ftsZ2* (lane #2 *f2*) (HV30 × pTA232-tele-0582anti#2 × pTA409-pfdx-HVO_0581-nat.t [*ftsZ2*]) and *cdrS*-*ftsZ2* (lane #2 *cdr-f2*) (HV30 × pTA232-tele-0582anti#2 × pTA409-pfdx-HVO_0582-HVO_0581-nat.t [*cdrS*-*ftsZ2*]). (A) Hybridization with a probe against HVO_B0192 and HVO_B0193, two genes that are upregulated in CRISPRi cells, confirmed upregulation (lane #2), and upregulation was even more prominent in CRISPRi cells complemented with *ftsZ2* only. Both genes are transcribed together into an approximately 1,077-nucleotide (Nt) mRNA. (B) Hybridization with a probe against HVO_0739, a gene that is downregulated in the CRISPRi strain, confirmed that the mRNA is downregulated in CRISPRi cells (lane #2). The mRNA is also downregulated in CRISPRi cells complemented with *ftsz2* (lane #2 *f2*). Complementation with both genes results in wild-type mRNA levels (lane #2 *cdr-f2*). The gene is transcribed into a monocistronic mRNA of about 1,052 nucleotides. Both membranes were also hybridized with a probe against the 16S rRNA (bottom).

10.1128/mBio.01416-21.7 TABLE S1 (A.I and A.II) Genes up- and down regulated in *cdrS-ftsZ2* CRISPRi cells. From 3,595 genes, transcripts were detected; altogether, 97 genes had significant expression differences during *cdrS-ftZ2* repression, with 35 genes upregulated (A.I) and 62 genes downregulated (A.II). Genes are sorted according to annotated functional category. The complete list of up- and downregulated genes can be found in Table S4A. All data have an adjusted *P* value of <0.005. TMD, predicted transmembrane domain. (A.I) Upregulated genes. The four upregulated genes HVO_B0193s2, HVO_B0193s, HVO_B0193, and HVO_B0192 (marked with an asterisk) might be part of an operon. HVO_B0192 and HVO_B0193 have already been shown to constitute an operon (see Fig. 3A). (A.II) Downregulated genes. (B) Proteins with significant changes in abundance. Proteins with significant changes in abundance from soluble (supernatant) and insoluble fractions, sorted according to annotated functional category. The full list of quantified proteins can be found in Table S4B. As a proportion of total protein-coding genes, an average of about 37% (1,499 proteins) proteome coverage was obtained. TMD, predicted transmembrane domain. For differential abundance data in the last two columns: On, proteins are only found in CRISPRi cells; Off, proteins are only found in wild-type cells; log_2_ ratio, log2 values; SN, supernatant fraction. Download Table S1, PDF file, 0.1 MB. Copyright © 2021 Liao et al. 2021 Liao et al. https://creativecommons.org/licenses/by/4.0/ This content is distributed under the terms of the Creative Commons Attribution 4.0 International license.

10.1128/mBio.01416-21.10 TABLE S4 (A) Complete list of up- and downregulated genes. (B) Complete list of regulated proteins. Download Table S4, XLSX file, 0.6 MB. Copyright © 2021 Liao et al. 2021 Liao et al. https://creativecommons.org/licenses/by/4.0/ This content is distributed under the terms of the Creative Commons Attribution 4.0 International license.

The transcriptome data confirmed repression of *cdrS* (log_2_ −2.5) and *ftsZ2* (log_2_ −2.6). In addition, the data revealed expression differences for 97 genes (with log_2_ fold changes lower and higher than −0.7 and +0.7, respectively). Thirty-five genes were upregulated (Table S1A), and 62 genes downregulated (Table S1A). Thirty genes encoding secreted, membrane, and cell surface proteins were differentially expressed (12 upregulated and 18 downregulated), suggesting significant changes to the cell envelope. Other upregulated genes included 1 involved in transport, 4 in transcription regulation (which could mediate some effects of *cdrS* repression), 2 in signal transduction, 2 in DNA maintenance and repair, 3 in metabolism, 1 in branched-side-chain amino acid biosynthesis, and 11 with unknown functions (Table S1A). Four downregulated genes were implicated in cell cycle and division, including *ftsZ1*, *ftsZ2*, *sepF*, and an *smc* (structural maintenance of chromosomes) homologue. Other downregulated genes are implicated in transport of branched-chain amino acids and sugars, transcription, cobalamin (vitamin B_12_) biosynthesis, amino acid metabolism (arginine/lysine), and general metabolism (Table S1A).

Since the CRISPRi approach represses both *cdrS* and *ftsZ2*, we next aimed to identify genes that are specifically regulated upon *cdrS* repression only, by complementing the CRISPRi strain with either the *ftsZ2* gene alone or the *cdrS-ftsZ2* genes together. For Northern blot analyses, we selected two genes that were found to be regulated in the *cdrS* CRISPRi transcriptome: the upregulated genes HVO_B0192-HVO_B0193 and the downregulated gene HVO_0739. Northern blots with RNAs from wild-type (HV30 × pTA232 × pTA409), *cdrS* CRISPRi (HV30 × pTA232-tele-0582anti#2 × pTA409), and complemented (HV30 × pTA232-tele-anti#2 × pTA409*ftsZ2* and HV30 × pTA232-tele-anti#2 × pTA409*ftsZ2-cdrS*) strains were hybridized with probes against the selected mRNAs (Fig. 3). Consistent with the transcriptome results, genes HVO_B0192-HVO_B0193 were found to be upregulated in the CRISPRi#2 strain complemented with *ftsZ2* only (Fig. 3). Likewise, HVO_0739 was confirmed to be downregulated in the CRISPRi cells complemented with only *ftsZ2*.

Taken together, the data indicated that upregulation of HVO_B0192-HVOB0193 and downregulation of HVO_0739 were due specifically to *cdrS* repression.

### Repression of *cdrS-ftsZ2* with CRISPRi causes multiple changes to the proteome.

We next compared the soluble and insoluble fractions of the wild-type and CRISPRi (*cdrS* CRISPRi#2) strains by quantitative proteomics. Previous proteome analyses of H. volcanii have shown that standard mass spectrometry techniques are biased against the detection of small proteins (20, 45). However, the CdrS protein was identified in two of the three wild-type replicates (with one peptide each), but with detection in only two replicates, it did not meet our criteria for listing in Table S1B (the detailed data set is provided in Table S4B). CdrS was not detected in any of the three CRISPRi strain replicates, consistent with repression of the *cdrS* gene. Thirty-four proteins were found to be more abundant or only found in CRISPRi cells, including 2 hypothetical transmembrane proteins, 2 involved in transport, 4 in translation, 5 in carbohydrate metabolism, 1 in central carbon metabolism (acetyl-CoA synthetase), 1 in arginine biosynthesis, 1 in lipid metabolism (isoprenyl diphosphate synthase), 3 in DNA replication and repair, 11 in general metabolism, and 4 with unknown functions (Table S1B). Conversely, 23 proteins were found to have lower abundance or were absent from the CRISPRi cells, including 6 transmembrane proteins, 2 involved in transport, one 30S ribosomal protein S15, 2 isocitrate lyase regulator-type (IclR-type) transcriptional regulators, 1 in carbohydrate metabolism, 2 in amino acid metabolism, 4 in general metabolism, 1 photolyase homologue (*phr1*) for DNA repair, 2 in cell division (FtsZ2 and SepF), and 2 with unknown functions (Table S1B). The changes in the abundance of proteins implicated particularly in metabolism, metabolite transport and regulation, and cell division suggest that CdrS might be involved in coordinating aspects of metabolism and growth with division.

### CdrS associates with a specific DNA motif *in vivo*.

**FIG 4:**
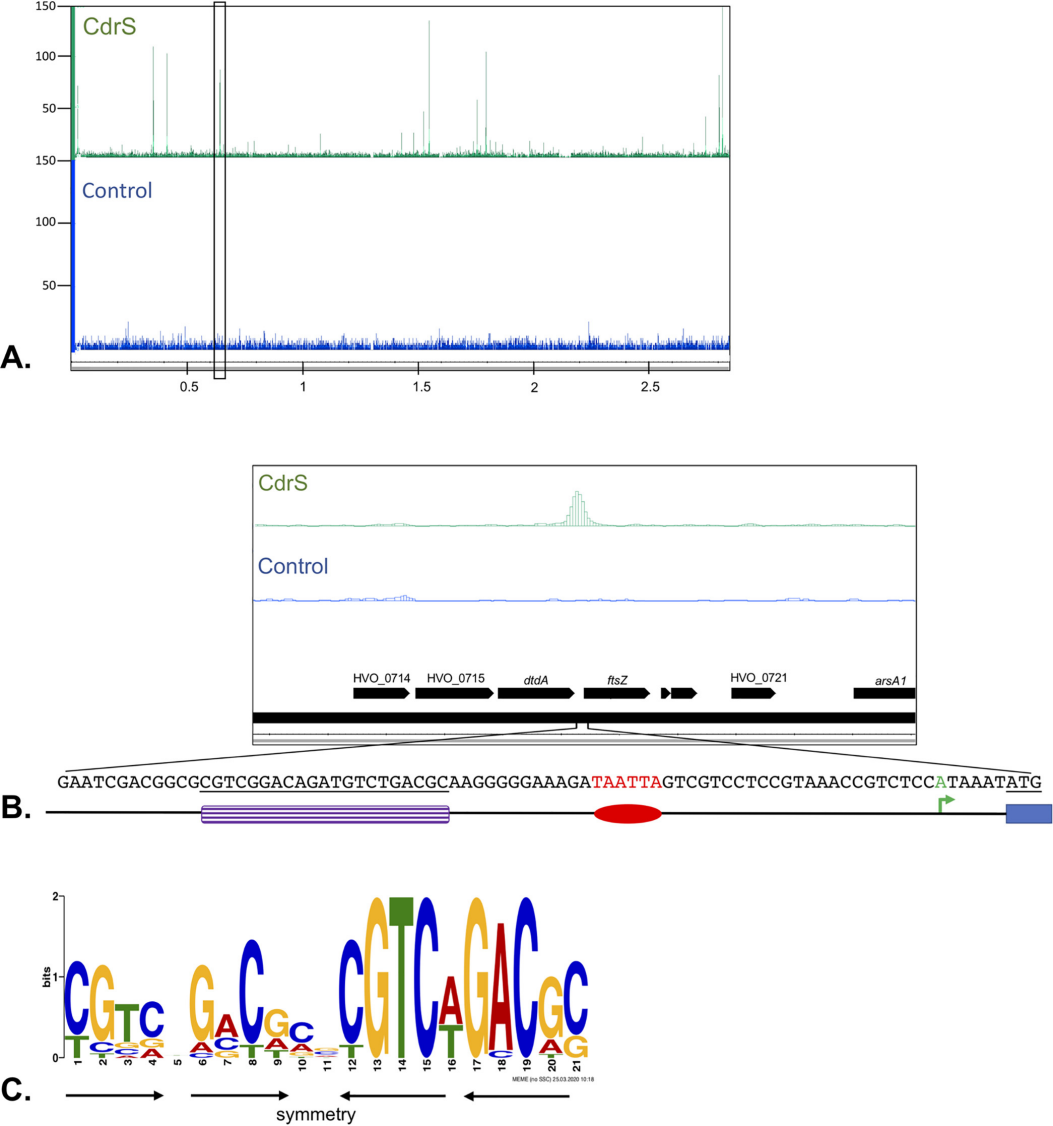
(A) Identification of the CdrS DNA binding motif. ChIP-Seq was employed to identify binding sites of CdrS. Control, ChIP-Seq analysis with the control sample (blue); CdrS, ChIP-Seq with the CdrS protein (green). CdrS binds to 18 locations in the genome. The ChIP-Seq data for the complete main chromosome (2.8 Mb) are shown. The chromosomal region highlighted by the black rectangle is shown enlarged in panel B. Read numbers are shown at the left; the chromosome coordinates are shown in Mb at the bottom. (B) CdrS binding site upstream from *ftsZ1*. The chromosomal region highlighted by the black rectangle in panel A is located upstream from the *ftsZ1* gene. Annotated genes are shown at the bottom; the sequence upstream from the *ftsZ1* gene is enlarged below the genome annotation. The CdrS binding motif is shown in purple, the promoter is shown in red, the TSS is shown in green, and the start of the gene (ATG) is underlined and shown as a blue box. (C) The conserved DNA binding motif identified using MEME-ChIP (68) shows notable symmetry (arrows), which might reflect the binding of CdrS multimer.

Since the predicted function for CdrS is a transcriptional regulator, we identified DNA binding sites of CdrS *in vivo* using chromatin immunoprecipitation-DNA sequencing (ChIP-Seq). Eighteen binding sites were revealed by ChIP-Seq (Table 1 and Fig. 4A), which allowed the identification of a specific binding site motif (Fig. 4C). For 15 of the 18 sites, the motif is located between 110 and 41 nucleotides upstream from the transcription start site (TSS) of a gene (Table 1). One binding site is located upstream from two closely spaced TSSs (−57 and −74 nucleotides upstream), two binding sites overlap a TSS, and for one binding site, no TSS is present under the standard growth conditions. Two binding sites were found in close proximity on opposite strands of two divergent genes, *hisB* (HVO_2986) and HVO_2987 (Fig. S2). CdrS binding sites were only detected on the main chromosome and not on the minichromosomes pHV1, pHV3, and pHV4.

**TABLE 1:** DNA binding sites identified for CdrS^*a*^

Downstream gene^*b*^	Annotation	Motif location (nt)^*c*^
HVO_0717 down	Cell division protein FtsZ1	−50
HVO_1660	Diadenylate cyclase (DacZ)	−47
HVO_1907	Cdc48d	−110
HVO_0850	Proteasome-activating nucleotidase (PanA)	−92
HVO_1562	20S proteasome beta subunit (PsmB)	−72
HVO_1885s down	RNA with unknown function	−41
HVO_0464	Threonine ammonia-lyase	−95
HVO_1691	PRC domain protein	−72
HVO_0398	Conserved hypothetical protein	−44
HVO_1611	Conserved hypothetical protein	−71
HVO_2968A	Conserved hypothetical protein	−42
HVO_1944	Conserved hypothetical protein containing a signal peptide	−72
HVO_2986 minus strand	Imidazoleglycerol-phosphate dehydratase	−76
HVO_2987 plus strand	Conserved hypothetical protein, containing a signal peptide and a transmembrane domain	−42
Between HVO_0027 and HVO_0029 on plus strand	Gene not annotated, binding site overlaps with TSS	0
Between HVO_1059 and HVO_1063 on minus strand	No TSS in this region	NA
Between HVO_1182 and HVO_1185 on plus strand	Gene not annotated, two TSSs downstream	−57 and −74
Between HVO_2907 and HVO_2910 on minus strand	Gene not annotated, binding site overlaps with TSS	0

a ChIP-Seq showed that CdrS binds to 18 different sites in the chromosome. For one location, the binding site is present on both strands (HVO_2986 and HVO_2987).

b Lists the gene located downstream from the target site and whether the gene is downregulated in the CRISPRi strain (“down”) (see Table S1A).

c Shows the distance from the identified binding motif (counting from the 10th nucleotide [nt] of the motif) to the transcription start site of each gene. NA, not applicable.

10.1128/mBio.01416-21.2 FIG S2 ChIP-Seq data from the region between the genes *hisB* (HVO_2986) and HVO_2987. Here, the CdrS binding motif is found on both strands, on the plus strand upstream from HVO_2987 and on the minus strand upstream from the *hisB* gene (HVO_2986). The control (C) is shown in blue at the top, and the ChIP-Seq sample with CdrS is shown in green. Read numbers are indicated at the left. Download FIG S2, PDF file, 0.01 MB. Copyright © 2021 Liao et al. 2021 Liao et al. https://creativecommons.org/licenses/by/4.0/ This content is distributed under the terms of the Creative Commons Attribution 4.0 International license.

**FIG 5:**
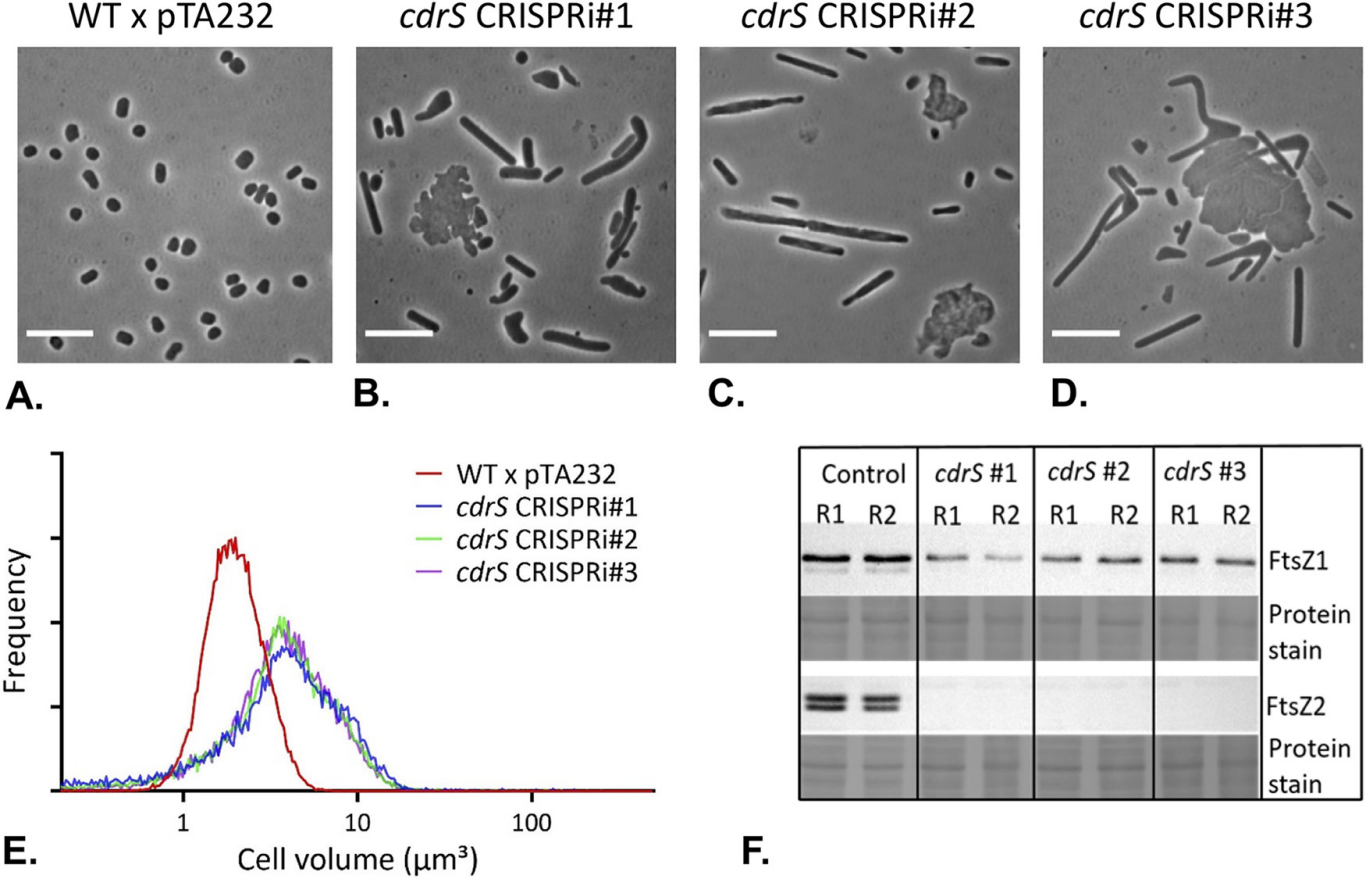
CRISPRi repression of *cdrS-ftsZ2* results in defects in cell division. (A to D) Phase-contrast images of the wild-type strain without crRNA expression, WT × pTA232 (HV35 × pTA232) (A), and three *cdrS* repression strains expressing the three different crRNAs, *cdrS* CRISPRi#1 (HV35 × pTA232-0582anti#1) (B), *cdrS* CRISPRi#2 (HV35 × pTA232-0582anti#2) (C), and *cdrS* CRISPRi#3 (HV35 × pTA232-0582anti#3) (D). All strains were sampled during steady mid-log-phase growth in Hv-MinTE medium supplemented with 50 μg/ml uracil and 0.04 mM l-tryptophan. Scale bars, 10 μm. (E) Coulter cytometry cell volume distributions obtained from the samples shown in panels A to D. Cell volume is shown on the *x* axis, and frequency (relative fraction of total cells) on the *y* axis. (F) Western blot analysis of FtsZ1 and FtsZ2 expression levels for control (HV35 × pTA232) and three *cdrS* repression strains (*cdrS* #1, HV35 × pTA232-0582anti#1; *cdrS* #2, HV35 × pTA232-0582anti#2; *cdrS* #3, HV35 × pTA232-0582anti#3). Total-protein prestaining of each membrane (with Ponceau S) is shown as a loading control. R1 and R2, two independent biological replicate protein samples. Data displayed are representative of at least two technical replicate experiments.

CdrS bound upstream from the *ftsZ1* gene (Fig. 4B), implicating CdrS in the regulation of a gene involved in cell division (16). According to the transcriptome data, the *ftsZ1* gene is moderately repressed in CRISPRi cells (HVO_0717; log_2_ fold change, −1.3) (Table S1A), suggesting that CdrS may activate *ftsZ1* transcription in wild-type cells. Proteome data of the CRISPRi strain showed an FtsZ1 abundance that appeared slightly lower than in the wild type (log_2_ fold change, −1.2), but with a *P* value of 0.13, it did not pass the parameter threshold (Table S4B). However, Western blot analyses with antibodies raised specifically against FtsZ1 and FtsZ2 were consistent with the above-described data; FtsZ2 was not detected in CRISPRi cells and FtsZ1 concentrations were reduced (Fig. 5F).

Another gene targeted by CdrS was the gene for diadenylate cyclase, which is essential and generates the signaling molecule c-di-AMP in H. volcanii (18). CdrS also binds upstream from the promoter for an RNA gene of unknown function, HVO_1885s. HVO_1885s was downregulated in CRISPRi cells, according to the transcriptome data (log_2_ fold change, −2.2) (Table S1A), and therefore might normally be activated by CdrS. Further target genes encode proteins that are involved in proteasome activity (Cdc48d [46], PanA [47, 48], and PsmB [47, 48]). In addition, CdrS binds to 7 genes encoding proteins and an RNA with unknown functions, as well as to 3 regions upstream from unannotated potential genes.

### Repression of *cdrS-ftsZ2* with CRISPRi has a severe effect on cell size and morphology.

Microscopic analyses revealed that the three CRISPRi strains showed substantial changes in cell size and morphology, featuring giant and misshapen plate-like cells, as well as long filamentous cells, during mid-logarithmic phase (Fig. 5B to D; Fig. S3). The giant cells are a hallmark of a cell division defect, since cells grow but division is delayed or fails, resulting in overgrowth. As H. volcanii can exist as rods or plate-shaped cells in culture, the filamentous and giant-plate cell types in the CRISPRi strains are expected to be the result of cell division defects in these two cell morphotypes (16). The three CRISPRi strains had very similar cell size defects compared to the wild-type cells (Fig. 5A to E), which were consistent throughout the growth phases (Fig. S3B) and showed similar cell morphology profiles (Fig. S4 and Table S2). Western blotting showed that repression of *cdrS* decreased FtsZ1 concentrations slightly and FtsZ2 levels drastically (Fig. 5F). The effect on FtsZ1 varied somewhat for the three CRISPRi strains (FtsZ1 was decreased ∼35% in *cdrS* CRISPRi#1, ∼69% in *cdrS* CRISPRi#2, and ∼76% in *cdrS* CRISPRi#3 compared to its level in the wild type), whereas FtsZ2 was strongly depleted in all cases (Fig. 5F). Together, these data implicate CdrS in the regulation of cell division and show that it has a direct or indirect influence on cell shape.

10.1128/mBio.01416-21.3 FIG S3 (A) Cell shape quantification analysis of *cdrS* repression during mid-log-phase growth in Hv-MinTE medium (plus 10 μg/ml uracil and 0.04 mM l-tryptophan [Trp]). Scatter plots of single-cell values for cell area (μm^2^) versus cell circularity for wild-type (HV35 × pTA232; *n* = 406), *cdrS* CRISPRi#1 (HV35 × pTA232-0582anti#1; *n* = 314), *cdrS* CRISPRi#2 (HV35 × pTA232-0582anti#2; *n* = 330), and *cdrS* CRISPRi#3 (HV35 × pTA232-0582anti#3; *n* = 173). The percentages of cells in the size and shape categories (see Materials and Methods) were as follows: *cdrS* CRISPRi#1, 20% filamentous, 5.7% giant-plate cell; *cdrS* CRISPRi#2, 30% filamentous, 3% giant-plate cell; and *cdrS* CRISPRi#3, 52.6% filamentous, 4.6% giant-plate cell. (B.A and B.B) Different growth stages and tryptophan concentrations did not induce different effects of *cdrS* CRISPRi on cell division defects. (B.A) Growth of four strains, including wild type (HV35 × pTA232), *cdrS* CRISPRi#1, *cdrS* CRISPR#2, and *cdrS* CRISPR#3, was performed in Hv-MinTE medium (plus 10 μg/ml uracil) under 0.04 mM, 0.08 mM, and 0.2 mM Trp (left vertical plane). Expression of the mRNAs for the Cascade complex proteins is regulated by the p*tnaA* promoter, which is induced by addition of tryptophan; more tryptophan results in more Cascade complexes. The cell volumes of four strains were analyzed under conditions of 0.04 mM Trp (red), 0.08 mM Trp (blue), and 0.2 mM Trp (green) at different growth time points of 15 h, 20 h, 25 h, and 40 h. (B.B) Phase-contrast images and Coulter cell volume analyses of wild-type, *cdrS* CRISPRi #1, *cdrS* CRISPRi#2, and *cdrS* CRISPRi#3 strains sampled at 25 h (log growth) under 0.2 mM Trp. All three *cdrS* CRISPRi strains showed similar cell division defects compared to growth under 0.04 mM Trp (see Fig. 5). Scale bars, 10 μm. The cell volumes of the three CRISPRi strains showed similar and larger size distributions compared to the wild-type. Frequency (*y* axis) is the relative fraction of total cells. Download FIG S3, PDF file, 1.2 MB. Copyright © 2021 Liao et al. 2021 Liao et al. https://creativecommons.org/licenses/by/4.0/ This content is distributed under the terms of the Creative Commons Attribution 4.0 International license.

10.1128/mBio.01416-21.4 FIG S4 Characterization of cell division phenotype upon complementation expression of *ftsZ2*, *ftsZ2-cdrS*, and *cdrS* in *cdrS* CRISPRi#1 and *cdrS* CRISPRi#2 backgrounds. Both the phase-contrast images and the corresponding Coulter cell volume analysis of *cdrS* CRISPRi#1 complementation strains (top) and *cdrS* CRISPRi#2 complementation strains (bottom) showed that the supplemental expression of both *ftsZ2* and *cdrS* restored cell division, whereas single complementation expression of *ftsZ2* or *cdrS* failed to rescue cell division. All the strains were sampled during steady mid-log-phase growth in Hv-MinTE medium supplemented with 0.04 mM l-tryptophan. Scale bars, 10 μm. Frequency (*y* axis) is the relative fraction of total cells. Download FIG S4, PDF file, 0.4 MB. Copyright © 2021 Liao et al. 2021 Liao et al. https://creativecommons.org/licenses/by/4.0/ This content is distributed under the terms of the Creative Commons Attribution 4.0 International license.

10.1128/mBio.01416-21.8 TABLE S2 Quantitative analysis of cell shape. The cell division mutants were quantified as four major distinct cell shapes: filaments (cell area ≥ 6.5 μm^2^, circularity ≤0.4), giant plate cells (cell area ≥6.5 μm^2^, circularity > 0.4); wild-type-like cells (cell area between 2 μm^2^ and 6.5 μm^2^); and cellular debris (cell area < 2 μm^2^). Analyzed were WT (WT × pTA232), *cdrS* repression (*cdrS* CRISPRi#1, -#2, and -#3) (see Fig. 5A), and overexpression (WT × *cdrS*) (see Fig. 7D) cells. Download Table S2, PDF file, 0.02 MB. Copyright © 2021 Liao et al. 2021 Liao et al. https://creativecommons.org/licenses/by/4.0/ This content is distributed under the terms of the Creative Commons Attribution 4.0 International license.

### Cell division defects are rescued by complementation with both *cdrS* and *ftsZ2*.

**FIG 6:**
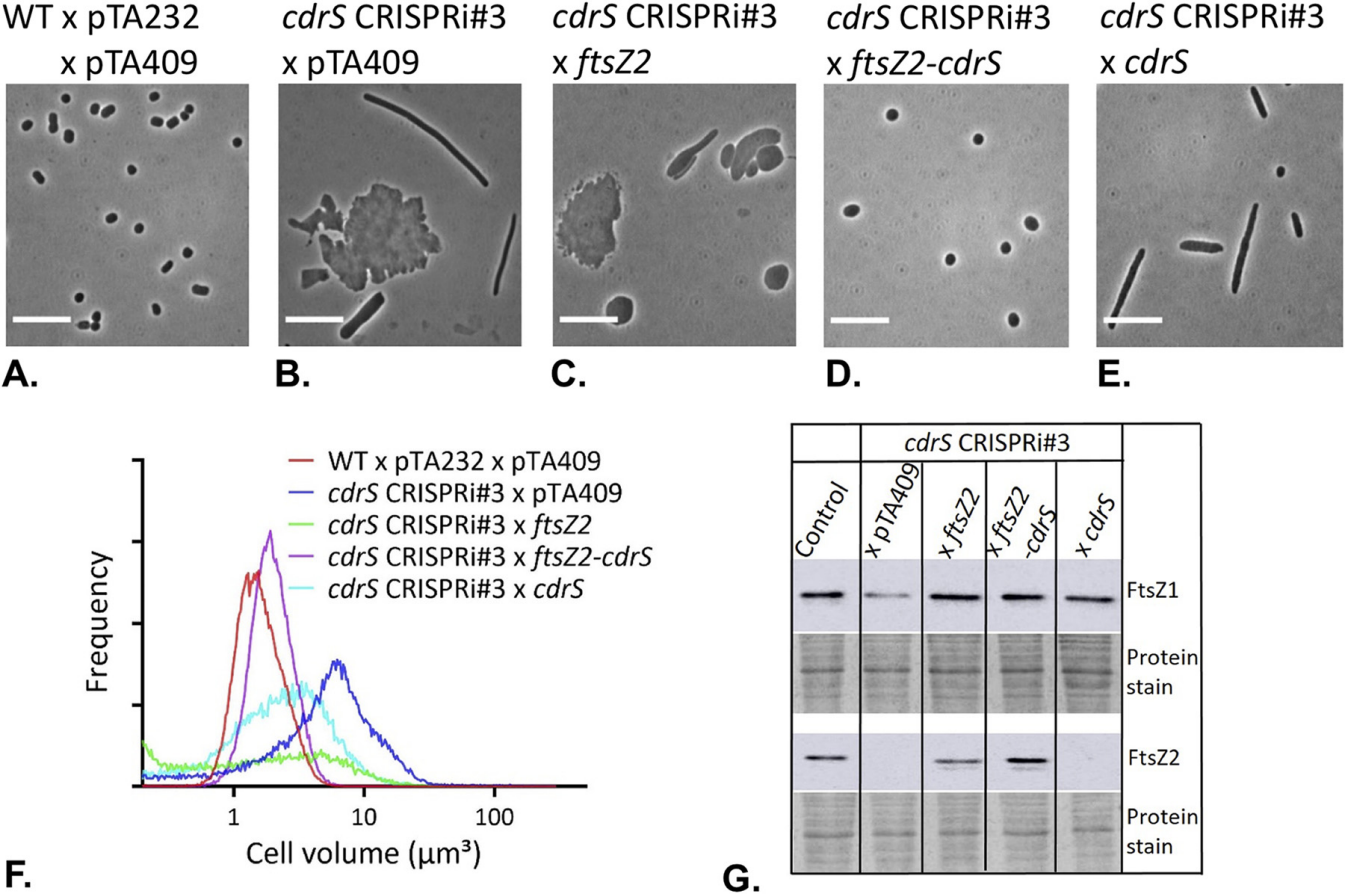
Complementation of CRISPRi strains. (A to E) Phase-contrast micrographs. Cell size in the CRISPRi#3 strain appeared normal with supplemental expression of both *ftsZ2* and *cdrS* but not during expression of *ftsZ2* or *cdrS* individually. Phase-contrast images of cells bearing wild-type vector only as the control for complementation (HV35 × pTA232 × pTA409) (A), *cdrS* CRISPRi#3 vector only (HV35 × pTA232-0582anti#3 × pTA409) (B), *ftsZ2* complementation of CRISPRi#3 cells (HV35 × pTA232-0582anti#3 × pTA409*ftsZ2*) (C), *ftsZ2*-*cdrS* double complementation of CRISPRi#3 cells (HV35 × pTA232-0582anti#3 × pTA409*ftsZ2-cdrS*) (D), and *cdrS* complementation of CRISPRi#3 cells (HV35 × pTA232-0582anti#3 × pTA409*cdrS*) (E). Scale bars, 10 μm. (F) Coulter cell volume analysis. Coulter cell volume analysis of the complementation effects of *cdrS* CRISPRi#3 in samples as listed in panels A to E. Frequency (*y* axis) is the relative fraction of total cells. (G) Western blot analysis of FtsZ1 and FtsZ2 expression levels for control (HV35 × pTA232 × pTA409), *cdrS* CRISPRi#3 vector only, *ftsZ2* complementation of *cdrS* CRISPRi#3, *ftsZ2-cdrS* double complementation of *cdrS* CRISPRi#3, and *cdrS* complementation of *cdrS* CRISPRi#3. Total-protein prestaining of each membrane (with Ponceau S) is shown as a loading control. Data displayed are representative of two independent experiments.

To determine whether the cellular effects we observed in the experiment whose results are shown in Fig. 5 were due to repression of *cdrS* and *ftsZ2* together or either one alone, we complemented the CRISPRi strain (*cdrS* CRISPRi#3) with plasmids expressing the *cdrS* gene alone, the *ftsZ2* gene alone, or both genes together. Only complementation with both genes together rescued the cell morphology and division defects (Fig. 6; Fig. S4). Western blotting showed that the complementation with *cdrS* alone did not restore FtsZ2 levels, and the FtsZ2 level was restored when *ftsZ2* was included on the complementation plasmid, as expected (Fig. 6G). These data suggest that CdrS is important for cell division independently of FtsZ2.

### Overexpression of *cdrS* induces cell size and morphological defects.

**FIG 7:**
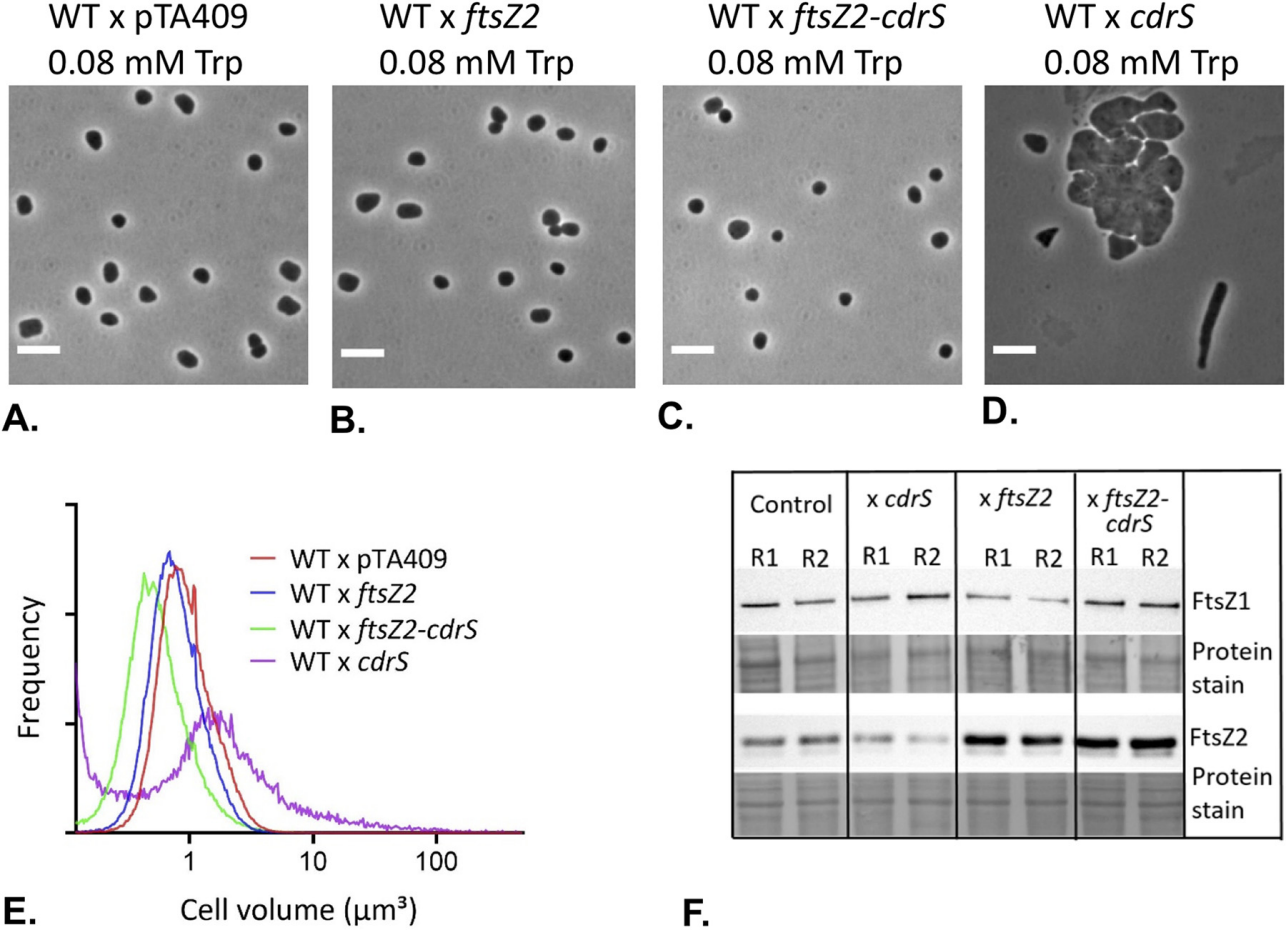
Overproduction of FtsZ2 and/or CdrS in wild-type background differentially affected cell division. (A to D) Phase-contrast micrographs. Wild-type (HV35 × pTA409) (A), FtsZ2 overexpression (HV35 × pTA409*ftsZ2*) (B), FtsZ2-CdrS double overexpression (HV35 × pTA409*ftsZ2-cdrS*) (C), and CdrS overexpression (HV35 × pTA409*cdrS*) (D). Compared to the wild type, both FtsZ2 single- and FtsZ2-CdrS double overexpression produced cells having wild-type morphology, whereas CdrS overexpression resulted in elongated/enlarged cells. Scale bars, 5 μm. (E) Coulter cell volume analysis. Cells overexpressing FtsZ2 produced cells with a slightly smaller size than the wild type, and FtsZ2-CdrS double overexpression produced significantly smaller cells, reflecting hyper-cell division, while CdrS overexpression resulted in significantly larger cells, indicating inefficient or misregulated cell division. Frequency (*y* axis) is the relative fraction of total cells. (F) Western blot analysis of FtsZ1 and FtsZ2 expression levels for control (HV35 × pTA409), CdrS overexpression, FtsZ2 overexpression, and FtsZ2-CdrS double overexpression. Total-protein prestaining of each membrane (with Ponceau S) is shown as a loading control. R1 and R2, two independent biological replicate protein samples. Data displayed are representative of at least two technical replicate experiments.

We next sought to identify any effects of overexpression of *cdrS*, *ftsZ2*, or both genes together from a constitutive strong promoter in a wild-type background. Interestingly, overexpression of *cdrS* alone had clear effects on cell size and morphology, showing some elongated (2.3%) cells and some large and misshapen cells (45%), as well as some wild-type-like cells (40.5%) (Fig. 7D and E; Fig. S5 and Table S2), consistent with misregulated division. In contrast, consistent with results using an inducible promoter (16), FtsZ2-only overexpression showed slightly smaller cells than the wild type (Fig. 7B and E; Fig. S5), termed hyperdivision. Furthermore, overexpression of both *cdrS* and *ftsZ2* together resulted in wild-type-like cells that had significantly smaller cell sizes than both the wild-type and *ftsZ2*-only overexpression strains (Fig. 7C and E; Fig. S5).

10.1128/mBio.01416-21.5 FIG S5 (A.A and A.B) Characterization of cell division phenotype upon overproduction of FtsZ2 and/or CdrS in HV35 background with different concentrations of l-tryptophan (Trp) (A.A) and in H26 background (Δ*pyrE2*) without Trp (A.B). (A.A) Phase-contrast images and corresponding Coulter cell volume analyses of wild-type control (HV35 × pTA409), *ftsZ2* overexpression (HV35 × *ftsZ2*), *ftsZ2*-*cdrS* overexpression (HV35 × *ftsZ2*), and *cdrS* overexpression (HV35 × *ftsZ2*) strains sampled in the Hv-MinTE medium (plus leucine, 50 μg/ml) with 0.04 mM Trp (top) or 0.2 mM Trp (bottom). The data showed that double *ftsZ2*-*cdrS* overexpression resulted in cells with wild-type morphology with significantly smaller cell size, while overexpression of single *cdrS* led to inefficient or misregulated division. Scale bars, 5 μm. Frequency (*y* axis) is the relative fraction of total cells. (A.B) Trp-independent *ftsZ2* and/or *cdrS* overexpression effect on cell division in H26. Phase-contrast micrographs (right) and corresponding coulter cell volume analysis of H26 control (H26 × pTA409), and *ftsZ2* overexpression, *ftsZ2*-*cdrS* overexpression, and *cdrS* overexpression in H26 background strains sampled during steady mid-log-phase growth in Hv-Cab medium. The overexpression of *ftsZ2* and/or *cdrS* in H26 without Trp in the medium showed similar effect in HV35 background with different concentrations of Trp in the medium. Scale bars, 5 μm. (B) Cell shape quantification analysis upon overproduction of FtsZ2 and/or CdrS in wild-type background. Scatter plots of single-cell values for cell area (μm^2^) versus cell circularity for wild type (HV35 × pTA409; *n* = 203), FtsZ2 overexpression (HV35 × pTA409*ftsZ2*; *n*= 173), FtsZ2-CdrS double overexpression (HV35 × pTA409*ftsZ2-cdrS*; *n* = 213), and CdrS overexpression (HV35 × pTA409*cdrS*; *n* = 131). Samples were taken from mid-log-phase growth in Hv-MinTE medium (plus leucine, 50 μg/ml) with 0.08 mM Trp (see Fig. 7). Download FIG S5, PDF file, 0.8 MB. Copyright © 2021 Liao et al. 2021 Liao et al. https://creativecommons.org/licenses/by/4.0/ This content is distributed under the terms of the Creative Commons Attribution 4.0 International license.

Western blot analysis of samples taken during *cdrS* overexpression indicated very little difference in the level of FtsZ2 (∼0.7-fold) compared to the level in the wild-type control, whereas the FtsZ1 level increased ∼1.7-fold (Fig. 7F), consistent with the CRISPRi results that suggest CdrS moderately promotes *ftsZ1* expression (Fig. 5). During *ftsZ2* overexpression, FtsZ1 levels were similar to the level in the wild type, whereas FtsZ2 levels were, as expected, substantially higher (∼2.3-fold) than in the wild type. FtsZ2 levels increased to ∼2.9-fold higher than in the wild-type in the *cdrS*-*ftsZ2* double overexpression strain (Fig. 7F), which might account for the additional stimulation of division observed in this strain (Fig. 7E). Finally, FtsZ1 increased ∼1.5-fold during double *cdrS*-*ftsZ2* overexpression, consistent with *cdrS* overexpression and CRISPRi repression results.

## DISCUSSION

Microscopic analyses of H. volcanii strains undergoing CRISPRi-mediated repression of the small gene *cdrS* (HVO_0582), supported by results obtained with complemented strains, showed that repression of *cdrS* expression alone had a severe impact on cell size and morphology; the most obvious defect at the cellular level appeared to be in the regulation or mechanism of cell division. This is supported by the finding that the *cdrS* gene was cotranscribed with a cell division gene, *ftsZ2*. ChIP-Seq revealed 18 CdrS binding sites, including one upstream from *ftsZ1*, another homolog involved in division (15). During CRISPRi-mediated repression of *cdrS*, *ftsZ1* was moderately downregulated, and strong downregulation was seen for other genes that might be involved in cell division, including HVO_0392 (encoding a homolog of the bacterial SepF division protein) and HVO_0739 (predicted membrane protein) (Table 2). Archaeal SepF was recently identified as an anchor for the division ring at the cytoplasmic membrane (49–52). The identified differential abundances of other proteins and genes involved in cell surface/membrane, transport, lipid metabolism, and carbohydrate metabolism (possible glycosylation) when *cdrS* is repressed suggest that CdrS may be a global regulator that controls division and other envelope-related functions in response to nutritional or metabolic changes.

**TABLE 2:** Genes and proteins affected in both the transcriptome and proteome of CRISPRi cells

Gene	Protein	Log_2_ fold change in^*a*^:	
		Transcriptome	Proteome
HVO_0581	FtsZ2	−2.6	−3.2
HVO_0392	Probable SepF protein	−2.2	−3.3
HVO_0739	Hypothetical protein	−3.3	Off

a Three genes were found to be downregulated in the transcriptome, and their protein products were found with lower abundance or “off” in the proteome.

Our results strongly suggest that CdrS functions as a transcription regulator. The majority of archaeal transcription factors have a helix-turn-helix (HTH) motif, while only a few contain the RHH domain (53). To our knowledge, similar small transcription factors have so far been described only for bacteria (40, 41). The binding location is usually an indicator of how a transcription factor acts: those activating transcription typically bind upstream from promoters (53), whereas those binding at or downstream from the promoter usually inhibit transcription by preventing recruitment of the RNA polymerase (53). CdrS binds upstream from the promoters of *ftsZ1* and HVO_1885s, which are downregulated in the CdrS depletion strain, consistent with CdrS normally acting as a transcriptional activator for these genes. CdrS also binds upstream from the *dacZ* gene that encodes diadenylate cyclase (DacZ), which synthesizes the second messenger molecule c-di-AMP. The *dacZ* gene is essential in H. volcanii, and overexpression of *dacZ* was lethal, indicating its central importance in cells and the need for tight regulation (18). The targets for c-di-AMP signaling in H. volcanii are currently unknown; we speculate that c-di-AMP regulation via CdrS might play a role in coordinating metabolic processes with the cell division or other envelope-related mechanisms. Consistent with this, our findings implicate CdrS as an activator of vitamin B_12_ (cobalamin) biosynthesis in H. volcanii (Table S1A), which has previously been noted to be under the regulatory network of the transcriptional regulator TrmB, a regulator of sugar metabolism in H. salinarum (54). Expression of the CdrS homolog in H. salinarum is itself regulated in response to oxidative stress (39). These results suggest that CdrS could take part in regulating division- and envelope-related functions in response to multiple global response pathways.

We also found CdrS binding sites upstream from three genes related to proteasome function (*cdc48d*, *panA*, and *psmB*). The gene *psmB* encodes the β subunit of the 20S proteasome in H. volcanii, which consists of proteins α1, β, and α2. The two proteasome-activating nucleotidases (PanA and PanB) are closely related to the regulatory particle AAA ATPases (Rpt) of eukaryotic 26S proteasomes (47, 48). Cdc48-like proteins appear to be universal among archaea and are linked to the function of the 20S proteasome in archaea (55). It has recently been shown in *Sulfolobus* that the activity of the proteasome is required for cells to divide and initiate the next round of DNA replication, thereby revealing a connection between the proteasome and cell division (56). It is possible that CdrS regulates a similar connection between them in H. volcanii. As the FtsZ-based cell division apparatus is dispensable in H. volcanii (15), the essential function of the *Haloferax* CdrS might lie in regulation of the essential DacZ and proteasome proteins. Interestingly, the CdrS homolog in H. salinarum (HbaCdrS; VNG0194H), which is also encoded in an operon together with FtsZ2, can be deleted (39); H. salinarum CdrS appears not to be involved in the regulation of DacZ and proteasome proteins, which might explain its nonessentiality.

We noted a general low correlation between the ChIP-Seq, proteome, and transcriptome data sets. A high correlation between the ChIP-Seq data and the omics data is not to be expected, since ChIP-Seq data reveal the binding activity of only CdrS, whereas the transcriptome and proteome results are due to repression of both CdrS and FtsZ2. Low correlation between transcriptome and proteome data has been widely reported in bacterial and eukaryotic cells and can be the consequence of technical limits to the detection of low-abundance mRNAs or proteins, as well as the involvement of additional layers of posttranscriptional regulation (57–60). In the case of CdrS, its influence on proteasome subunit transcription could have knock-on influences in the proteome beyond transcriptional control. Our results are consistent with a hypothesis that there is substantial posttranscriptional regulation in the regulatory systems involving CdrS, and this may extend to other regulatory pathways in H. volcanii.

In members of the *Haloarchaea*, the function of CdrS appears to be conserved in relation to the regulation of cell division (39). Our combined results suggest that CdrS is part of a regulatory network and controls the cell division apparatus and other downstream gene products in response to several conditions or stresses and via other transcription regulators. CdrS-mediated regulation of division might thereby play an important role in maintaining archaeal cell size homeostasis in coordination with metabolism or by triggering changes in cell size or morphology in response to conditions or stress.

## MATERIALS AND METHODS

### Strains and growth conditions.

The strains, plasmids, and oligonucleotides used are listed in Table S3. E. coli strains DH5α (Invitrogen, Thermo Fischer Scientific, Waltham, MA, USA) and GM121 (46) were grown aerobically at 37°C in 2YT medium (74).

10.1128/mBio.01416-21.9 TABLE S3 (A) Strains. Strains used in this study are listed. (B) Plasmids. Plasmids used in this study are listed. (C) Oligonucleotides. Oligonucleotides used in this study are listed. Download Table S3, PDF file, 0.01 MB. Copyright © 2021 Liao et al. 2021 Liao et al. https://creativecommons.org/licenses/by/4.0/ This content is distributed under the terms of the Creative Commons Attribution 4.0 International license.

H. volcanii strains HV30 and HV35 (44) with plasmids were grown aerobically at 45°C and 200 rpm in Hv-Min medium or Hv-Ca medium (61, 62) or in media modified to contain an expanded trace element and vitamin solution, which are referred to as Hv-MinTE (this study) or Hv-Cab (15, 16). Where necessary, the medium was supplemented with uracil (10 μg/ml or 50 μg/ml as indicated) (for a Δ*pyrE2* strain), leucine (50 μg/ml) (for a Δ*leuB* strain), and l-tryptophan with the indicated concentration (for a Δ*trpA* strain). Tryptophan was also added to media to control gene expression of the *cas* genes coding for proteins Cas5 to -8b via the tryptophan-inducible promoter (p*tnaA*). Unless otherwise indicated, cultures were generally maintained in steady logarithmic growth (optical density at 650 nm [OD_650_] of <0.8) for at least 2 days prior to sampling (OD_650_ = 0.2 to 0.8) for analysis. Data displayed are representative of at least three biological replicate experiments.

### Strains and plasmid construction. (i) Generation of strain HV35.

To generate a strain with inducible *cas* gene expression, the gene cluster *cas6b*, *cas8b*, *cas7*, and *cas5* was cloned downstream from the tryptophan-inducible promoter p*tnaA* and upstream from the terminator t*syn* into the pTA131 plasmid that contained the up- and downstream regions of CRISPR locus C, yielding pTA131-Cup-p.tnaA-cas6b8b75-t.syn-Cdo. Strain HV32 (43) was transformed with the plasmid pTA131-Cup-p.tnaA-cas6b8b75-t.syn-Cdo to mediate integration of the plasmid into the genome, replacing CRISPR locus C. Pop-in candidates were plated on medium containing 5-fluoroorotic acid (5-FOA) to select for pop-out clones. *cas* gene insertion candidates were verified by Southern blotting (Fig. S6A). Ten micrograms of genomic DNA was digested with SacII, and fragments were separated on a 0.8% agarose gel. DNA fragments were transferred to a nylon membrane (Hybond-N^+^; GE Healthcare, Dornstadt, Germany) by capillary blotting. Two PCR products (termed Cup and cas8) with sizes of 305 bp (Cup) and 399 bp (cas8) were used as hybridization probes. Fragment Cup was amplified using primers CdelupKpnI and CdelupiEcoRV, and fragment cas8 was amplified using primers 5-HindIII-Cas8 and 8R126A#2. Probes were labeled using [α-^32^P]dCTP and the DECAprime II DNA labeling kit (Thermo Fisher Scientific). Both hybridization and detection of the membranes were carried out as described in the manufacturer's protocol. The strain resulting from this *cas* gene integration with tryptophan-inducible promoter p*tnaA* was termed HV35.

10.1128/mBio.01416-21.6 FIG S6 (A.A and A.B) Southern blot analysis of strain HV35. To verify the *cas* gene insertion, genomic DNA from HV32 and four potential HV35 clones was isolated, and 10-μg amounts were digested by SacII. After separation of DNA fragments on a 0.8% agarose gel, fragments were transferred by capillary blotting to a nylon membrane (Amersham Hybond-N^+^; GE Healthcare) and fixed on the membrane by UV cross-linking. (A.A) Hybridization with a probe against the upstream region. Expected signals were 564 bp for HV32, and in the case of *cas* gene integration in HV35, the expected size was 1,155 bp. (A.B) Hybridization with a probe against *cas8b*. Since the probe cas8 binds to the *cas8b* gene, no signal was expected for HV32; HV35 strains showed the expected signal of 1,104 bp. Genomic locations are shown schematically to the side. (B) PCR analysis of all the *cdrS* CRISPRi and complementation strains to confirm the presence of the *cdrS* targeting spacer. The genomic DNA was extracted from the strains tested, including wild-type vector only (HV35 × pTA232), all three *cdrS* CRISPRi strains, and *ftsZ2*, *cdrS*, and double *ftsZ2-cdrS* complementation strains in the corresponding *cdrS* CRISPRi strains, and used as the template for the PCR to verify the presence of the *cdrS* crRNA spacer in the cell which directed the CRISPR knockdown effect on *cdrS*. The forward primers used for PCR were located within the *cdrS* crRNA spacer, and the reverse primers were universal primers pUC13/M13 Rev located ∼160 bp away from the downstream repeat motif in the mini-CRISPR array plasmid (see Fig. S1). The PCR analysis showed the presence of the *cdrS* crRNA spacer in all the *cdrS* CRISPRi strains and the corresponding complementation strains tested, suggesting that they did not escape CRISPRi-mediated editing via recombination between the repeat sequences flanking the crRNA spacer on the plasmid. DNA ladder to right, 100-bp DNA Ladder (NEB); DNA ladder to left, MassRuler DNA ladder mix (Thermo Fisher Scientific). Download FIG S6, PDF file, 1.0 MB. Copyright © 2021 Liao et al. 2021 Liao et al. https://creativecommons.org/licenses/by/4.0/ This content is distributed under the terms of the Creative Commons Attribution 4.0 International license.

### (ii) Attempt to generate a *cdrS* deletion strain.

To delete the *cdrS* gene, the gene was amplified with 500-bp flanking regions by using primers HVO_0582-UP and HVO_0582_DO. The resulting PCR product was ligated into pTA131 (digested with EcoRV), resulting in plasmid pTA131-up-HVO_0582-do. Inverse PCR on pTA131-up-HVO_0582-do with primers iPCR_HVO 0582_UP and iPCR_HVO_0582_DO_NEU deleted the gene HVO_0582. After ligation of the resulting PCR product, the plasmid pTA131-up-ΔHVO_0582-do was obtained. The wild-type strain H119 was transformed with pTA131-up-ΔHVO_0582-do to generate a knockout strain with the pop-in/pop-out method of Bitan-Banin et al. (42). Pop-in clones were obtained and confirmed via colony PCR with primers HVO_0582_UP and HVO_0582_DO. To obtain a pop-out strain, 409 pop-in clones were screened with PCR with primers HVO_0582_UP and HVO_0582_DO. All clones still contained the HVO_0582 gene, suggesting that HVO_0582 is essential.

### (iii) Plasmid pTA231-pfdx-HVO_0582NFlag used for ChIP-Seq.

Primers 5′-HVO_0582-HindIII and 3′-HVO_0582-XbaI were used for amplification of the gene *cdrS* (HVO_0582) using genomic DNA from H. volcanii strain H119. The DNA fragment obtained was digested with HindIII and XbaI and ligated into pTA927 (digested with HindIII and XbaI) to yield pTA927-ptna-HVO_0582NFlag. This plasmid was digested with NdeI and XbaI, and the resulting fragment was ligated into pTA231-pfdx (digested with NdeI and XbaI), resulting in pTA231-pfdx-HVO_0582NFlag.

### (iv) Plasmids pTA232-tele-anti#1 to -#3.

Plasmids expressing the crRNAs from a terminator element (tele)-containing precursor were generated by inverse PCR with pMA-telecrRNA (44, 63) as the template. The primers used (anti#1 fw, anti#1 rev/anti#2 fw, anti#2 rev/anti#3 fw, and anti#3 rev) replace spacer 1 of locus C with spacer anti#1, anti#2, or anti#3 against HVO_0582. Plasmids pMA-tele-anti#1, -#2, and -#3 comprise the crRNA gene (8-nucleotide 5′ handle, spacer, 22-nucleotide 3′ handle) flanked by terminator elements. Plasmids were digested with KpnI and BamHI, and the resulting fragment was cloned into pTA232 (61) digested with the same enzymes, resulting in plasmids pTA232-tele-anti#1, -#2, and -#3.

### (v) Plasmids pTA232-0582anti#1 to -#3.

Plasmids expressing the crRNA with flanking repeats were ordered (GeneArt, Thermo Fisher Scientific) as pMK-RQ-0582anti#1, -2#, and -#3. They contained a synthetic CRISPR locus, which comprises the leader of locus C, one spacer flanked by repeats, and a synthetic terminator. After digesting the plasmids with BamHI and KpnI to excise the entire locus, purified inserts were ligated into pTA232 digested with the same enzymes, yielding plasmids pTA232-0582anti#1, -2#, and -#3.

### (vi) Construction of the complementation plasmids.

Plasmids for complementation were generated via amplification of gene HVO_0581 or HVO_0582 or of both HVO_0582 and HVO_0581, including the natural terminator, using primers 5′-HVO_0582-NdeI, 3′-HVO_0582-HindIII, 5′-HVO_0581-NdeI, 3′-HVO_0581-nat.t-ApaI, and 5′-HindIII-nat.t. The PCR fragments were ligated into pBlue (digested with EcoRV), and the resulting plasmids were digested with NdeI and ApaI to isolate the inserts, which were ligated into plasmid pTA409-pfdx (digested with the same enzymes) to obtain the complementation plasmids pTA409-fdx-HVO_0581-nat.t, pTA409-fdx-HVO_0582-nat.t, and pTA409-fdx-HVO_0582-HVO_0581-nat.t.

### Gene repression with CRISPRi.

In *Haloferax*, two CRISPRi approaches that are both effective can be employed (44). In one approach, the crRNA gene is expressed between two terminator elements that are processed by the cellular proteins RNase P and tRNase Z to release the mature crRNA. For this approach, *Haloferax* strain HV30 is used. In a second approach, the crRNA is expressed as part of a short synthetic CRISPR locus that is processed by Cas6b to generate the mature crRNA; here, *Haloferax* strain HV35 is used. For microscopic analyses of cell morphology and growth experiments, the crRNAs were encoded in a synthetic CRISPR locus and expressed in HV35. For all other experiments, the crRNAs were produced via the Cas6b-independent mechanism (63) in HV30. This strain has had the *cas3* and *cas6b* genes deleted, ensuring that DNA bound by the Cascade complex is not degraded by Cas3 and endogenous crRNAs are not produced by Cas6b cleavage (43).

For repression of *cdrS* (HVO_0582), strain HV35 was transformed with the unmethylated (obtained via passage through E. coli GM121) CRISPR knockdown plasmid pTA232-0582-anti#1, pTA232-0582-anti#2, or pTA232-0582-anti#3, selecting the transformants on Hv-Min agar medium with uracil (10 μg/ml or 50 μg/ml) and tryptophan (0.04 mM or 0.2 mM) as indicated. Single colonies were streaked on the same medium, and colonies were screened by PCR to identify the presence of the crRNA spacer (Fig. S6B) and named *cdrS* CRISPRi#1, *cdrS* CRISPRi#2, and *cdrS* CRISPRi#3. Since homologous recombination can happen between the repeats and thereby delete the crRNA spacer, it was important to test the strains with PCR to confirm the presence of the complete crRNA gene in the plasmids. For the complementation test, strain HV35 was cotransformed with the unmethylated CRISPR knockdown plasmid (pTA232-0582-anti#1, pTA232-0582-anti#2, or pTA232-0582-anti#3) and the unmethylated expression plasmid (pTA409 as a control, pTA409-fdx-HVO_0581-nat.t, pTA409-fdx-HVO_0582-nat.t, or pTA409-fdx-HVO_0582-HVO_0581-nat.t). Selection for transformants containing two plasmids was achieved by growth on Hv-MinTE agar medium with l-tryptophan (0.04 mM). Single colonies were streaked on the same medium, and colonies were screened by PCR to identify the presence of the gene for the crRNA spacer (Fig. S6B).

For the overexpression test, strain HV35 was transformed with the unmethylated expression plasmid pTA409 (as a control), pTA409-fdx-HVO_0581-nat.t, pTA409-fdx-HVO_0582-nat.t, or pTA409-fdx-HVO_0582-HVO_0581-nat.t, followed by selecting the transformants on Hv-Cab agar medium with l-tryptophan (0.04 mM or 0.2 mM). The overexpression effect was also tested in H. volcanii strain H26 (Δ*pyrE2*) by selecting the transformants on Hv-Cab agar medium.

### Growth experiments.

Cells were grown aerobically with shaking (200 rpm) at 45°C in Hv-MinTE medium with the addition of tryptophan (0.08 mM) and uracil (50 μg/ml). Cell growth was monitored by measurement of the OD_650_. For each strain, three biological replicates were prepared. Doubling times *d* for strains were calculated as follows: the natural logarithm of 2 was divided by the growth rate μ (*d* = ln 2/μ). The growth rate μ itself is calculated through the natural logarithm of the values of the time range divided by the time range {μ = [ln *x*(*t*) – ln *x*(*t*_0_)]/(*t* – *t*_0_)}.

### Light microscopy.

For most phase-contrast microscopy, a 2-μl sample of culture was placed on an agarose pad prepared by dropping ∼50 μl of 1% agarose containing 18% buffered saltwater (BSW; includes calcium, 30% BSW stock contains 240 g NaCl, 30 g MgCl_2_ . 6H_2_O, 35 g MgSO_4_ . 7H_2_O, 7 g KCl, 5 ml 1 M CaCl_2_, and 20 ml 1 M Tris-Cl, pH 7.5, per liter) onto a glass slide at room temperature, and a clean 22- by 50-mm number 1.5 glass coverslip was placed on top. Images were acquired using a 100×/1.4 numeric aperture (NA) oil immersion objective and phase-contrast optics using a Zeiss Axioplan microscope (Carl Zeiss, Oberkochen, Germany).

### Coulter cell volume analysis.

Culture samples were diluted (1:100 or 1:1,000) with 0.2-μm-filtered 18% BSW and were analyzed with a Multisizer 4 Coulter counter (Beckman Coulter, Indianapolis, IN, USA) equipped with a 30-nm-aperture tube, calibrated with a 2-μm latex bead standard (Beckman Coulter) diluted in 18% BSW as the electrolyte. Runs were completed in volumetric mode (100 μl), and current set to 600 μA and gain to 4.

### Cell shape quantification.

Microscope images were analyzed using ImageJ 1.53c. Phase-contrast images were first smoothed using a Gaussian filter followed by a rolling-ball background subtraction. Individual objects (cells) were then identified by optimized thresholding. Parameters for detection were adjusted, and touched cells were manually split. The holes in the binary objects were filled by using a hole-filling operation. Cell shape parameters were determined by using the “Analyze particles” function. The minimum cell size was 0.2 μm^2^, and the edged objects were excluded. The circularity of each cell was calculated as the percentage of cell area to the minimal circle area that completely contains the cell outline (15), using a custom macro implemented in ImageJ (16). The cell division mutants were quantified as four distinct cell shapes, as follows: filaments (cell area ≥ 6.5 μm^2^ and circularity ≤ 0.4), giant plate cells (cell area ≥ 6.5 μm^2^ and circularity > 0.4), cellular debris (cell area ≤ 2 μm^2^), and wild-type-like cells (cell area between 2 μm^2^ and 6.5 μm^2^).

### Northern blot analysis. (i) For CRISPRi strains.

RNA was isolated from strains HV30 × pTA232-tele-0582anti#1, -#2, and -#3 and wild-type strain HV30 × pTA232 as described before (64). Ten micrograms of RNA was separated on a denaturing 8% polyacrylamide gel or a denaturing 1% agarose gel and transferred to a nylon membrane (Hybond-N^+^ membrane or Pall membrane). For hybridization experiments, radioactively labeled PCR probes against the desired targets were generated using the DECAprime II DNA labeling kit and [α-^32^P]dCTP (Life Technologies, Thermo Fisher Scientific). Membranes were subsequently incubated with the labeled PCR fragments.

To quantify the repression efficiency, Northern blotting membranes were exposed to imaging plates and analyzed with the Amersham Typhoon biomolecular imager and ImageQuantTL software. Signals were compared with the signals for the 16S rRNA used as a loading control. The amount of RNA signals detected for the wild-type controls was set to 100%. Northern blot analyses were done in triplicates.

### (ii) For complemented strains.

The *cdrS* CRISPRi strain (expressing crRNA#2) was complemented with either the single gene HVO_0581 (*ftsZ2*) or with the complete operon HVO_0582-HVO_0581. Transformed strains were grown in minimal medium supplemented with tryptophan (0.25 mM) and harvested in exponential phase (OD_650_ ≈ 0.4 to 0.52). RNA was isolated as described before (64), separated on a 1% agarose gel, and transferred onto a nylon membrane (Pall membrane). For hybridization experiments, radioactively labeled PCR probes against the selected targets were generated using the DECAprime II DNA labeling kit and [α-^32^P]dCTP (Life Technologies, Thermo Fisher Scientific). Membranes were subsequently incubated with the labeled PCR fragments against the mRNA of the cluster HVO_B0192-HVO_B0193 and against the mRNA of HVO_0739.

### ChIP-Seq. (i) Preparation of the samples.

The ChIP-Seq analysis was performed with the FLAG-tagged CdrS and a control with three replicates each. The HV35 × pTA231-pfdx-HVO_0582NFlag culture was grown in Hv-Ca medium supplemented with uracil (50 μg/ml) to an OD_650_ of 0.7 to 0.85 (replicate 1, 0.74; replicate 2, 0.72; replicate 3, 0.85). Cross-linking was done with a final concentration of 0.5% (vol/vol) formaldehyde for 20 min at room temperature under constant shaking. After 20 min, the reaction was stopped by the addition of glycine to a concentration of 0.25 M. Cells were harvested at 4°C and 9,800 × *g* for 20 min and washed twice with enriched phosphate-buffered saline (PBS). Cells were resuspended in the appropriate volume (*V*) of lysis buffer (*V*_culture_ × OD_650_/45 = ml lysis buffer). After ultrasonification using a Branson Sonifier, the solution was centrifuged at 15,500 × *g* and 4°C for 1 h, yielding the S15 protein fraction. The DNA was fragmented to an average length of 200 to 500 bp with ultrasonification for 60 min with a duty cycle of 50%, followed by a RNA digestion with RNase A for 30 min at 37°C. For the control, 10% of the S15 extract was removed and later processed in the same way as the purified protein-DNA complex was. The FLAG-tagged protein-DNA complex was purified via affinity chromatography using anti-FLAG M2 affinity gel (Sigma-Aldrich, Taufkirchen, Germany). The purified protein-DNA complexes and the control were incubated at 95°C for 40 min for reversal of the cross-linking. DNA samples were subsequently incubated with RNase A and proteinase K for 20 min at 37°C. After a phenol-chloroform extraction, DNA was precipitated and resolved in 10 μl DNase-free water, and the concentration was measured with a NanoDrop photometer.

### (ii) Library preparation and sequencing.

DNA library preparation was carried out according to the manufacturer’s protocol using the NEXTflex ChIP-Seq kit (NOVA-5143-01) (PerkinElmer, Hamburg, Germany) and the NEXTflex ChIP-Seq barcodes (NOVA-514122) (PerkinElmer). DNA libraries were pooled to 4 nM and sequenced using an Illumina MiSeq.

### (iii) Bioinformatics analyses.

In order to remove sequencing errors, a quality control was applied to the sequenced reads in FASTQ files using the FastQC tool (https://www.bioinformatics.babraham.ac.uk/projects/fastqc/), and then the TrimGalore tool (https://www.bioinformatics.babraham.ac.uk/projects/trim_galore/) was used to remove adapters and perform read quality trimming. The reads were then aligned to the main chromosome and the three plasmids pHV1, -3, and -4 via Bowtie2 (65). After that, we computed the correlation between read counts in different regions for all samples using deeptool (66). Next, we identified enriched binding sites (peaks) using MACS2 callpeak (67). Finally, the MEME-ChIP (68) tool was used for discovering motifs in the peak regions.

### Transcriptome analyses.

Strains HV30 × pTA232-tele-anti#2 and HV30 × pTA232 were grown in Hv-MinTE medium supplemented with tryptophan (0.25 mM) and uracil (50 μg/ml) to exponential phase (OD_650_ = 0.42 to 0.47), and experiments were done in triplicates. RNA was isolated as described for Northern blot analyses and sent to Vertis Biotechnologie AG (Munich, Germany) for cDNA synthesis and sequencing. From the total RNA samples, rRNA molecules were depleted using the Ribo-Zero rRNA removal kit for bacteria (Illumina, San Diego, CA, USA). The ribodepleted RNA samples were first fragmented using ultrasound (4 pulses of 30 s each at 4°C). Then, an oligonucleotide adapter was ligated to the 3′ end of the RNA molecules. First-strand cDNA synthesis was performed using Moloney murine leukemia virus (M-MLV) reverse transcriptase and the 3′ adapter as the primer. The first-strand cDNA was purified, and the 5′ Illumina TruSeq sequencing adapter was ligated to the 3′ end of the antisense cDNA. The resulting cDNA was PCR amplified to about 10 to 20 ng/μl using a high-fidelity DNA polymerase. The cDNA was purified using the Agencourt AMPure XP kit (Beckman Coulter). For Illumina sequencing, the cDNAs were pooled in approximately equimolar amounts. The library pool was fractionated in the size range of 200 to 550 bp using a differential cleanup with the Agencourt AMPure kit. The cDNA pool was sequenced on an Illumina NextSeq 500 system using a 75-bp read length.

### Bioinformatics analyses.

Quality control was applied to the sequenced reads in FASTQ files using the FastQC tool (https://www.bioinformatics.babraham.ac.uk/projects/fastqc/), and then the TrimGalore tool (https://www.bioinformatics.babraham.ac.uk/projects/trim_galore/) was used to remove adapters and perform read quality trimming. For analysis of transcriptome samples, reads were aligned to the main chromosome and minichromosomes (pHV1, pHV3, and pHV4) using Bowtie2 (65). Differential gene expression analysis was performed using DESeq2 (69) with default settings. We considered the genes with an adjusted *P* value of less than or equal to 0.005 to be significantly differentially expressed. With this cutoff, we allowed less than 1% of the false positives from the significantly differentially expressed genes. The complete output is shown in Table S4A.

### Proteome analyses.

Strains were grown as described for transcriptome analyses. After centrifugation, cells were resuspended in 18% salt water supplemented with 0.1 mM phenylmethylsulfonyl fluoride (PMSF), 1 mM benzamidine, 1 μg/ml pepstatin A, 1 μg/ml leupeptin, and 10 mM dithiothreitol (DTT). After ultrasonification with a 50% duty cycle for 3 min, taurodeoxycholate was added to a final concentration of 0.006%. The suspension was subsequently incubated for 30 min at 4°C, followed by ultracentrifugation at 30,000 × *g* for 45 min at 4°C. The supernatant and the pellet (which was resuspended with 50 mM Tris-HCl buffer [pH 7]) were incubated with 20 μl DNase I (RQ1), 0.2 μl exonuclease III, and 0.5 μl RNase A for 1 h at 4°C with gentle shaking. Soluble proteins from the supernatant fraction were precipitated with acetone. Insoluble proteins were purified with StrataClean beads (Agilent, Santa Clara, CA, USA).

For analysis of the soluble protein fraction, 50 μg protein was reduced with 2.5 mM TCEP (Tris-[2-carboxyethyl]phosphine hydrochloride; Invitrogen, Thermo Fisher Scientific) at 65°C for 45 min before thiols were alkylated in 5 mM iodoacetamide (Sigma-Aldrich) for 15 min at 25°C in the dark. For protein digestion, trypsin (Promega, Walldorf, Germany) was added in an enzyme-to-substrate ratio of 1:100 before incubation at 37°C for 14 h. For digestion of proteins in the insoluble fraction, 20 μl StrataClean beads (Agilent), which bound 20 μg protein, were incubated with 2.5 mM TCEP at 65°C for 45 min before 5 mM iodoacetamide was added. Samples were incubated for 15 min at 25°C in the dark and subsequently digested with trypsin (enzyme-to-substrate ratio of 1:100) for 14 h at 37°C. To ensure the complete generation and removal of peptides from the beads, further trypsin (200 ng) was added and incubation at 37°C was prolonged for additional 3 h. Peptides were eluted from the beads by incubation in a sonication bath for 2 min and transfer of the supernatant to a new vial. The beads were then washed sequentially with 100 μl solvent A (0.1% acetic acid in water) and 100 μl 60% solvent B (0.1% acetic acid in acetonitrile diluted in solvent A). After each washing step, peptides were again eluted by sonication and supernatants were collected in the same vial as the initial elution. Subsequently, peptides were dried by evaporation of the solvents and suspended in 10 μl solvent A before mass spectrometry (MS) analysis.

For MS analysis, peptides were loaded on an EASY-nLC 1000 system (Thermo Fisher Scientific) equipped with an in-house-built 20-cm column (inner diameter, 100 μm; outer diameter, 360 μm) filled with ReproSil-Pur 120 C_18_-AQ reversed-phase material (3-μm particles; Dr. Maisch GmbH, Ammerbuch-Entringen, Germany). Elution of peptides was executed with a nonlinear 180-min gradient from 1% to 99% solvent B (0.1% [vol/vol] acetic acid in acetonitrile) with a flow rate of 300 nl/min injected online into an Orbitrap Q Exactive (Thermo Fisher Scientific) in data-independent acquisition (DIA) mode. In each DIA cycle, one survey scan at a resolution of *R* = 70,000 at *m/z* 200, 120-ms maximal injection time (IT), 3 × 10^6^ automatic gain control target, and mass range of 300 to 1,250 was obtained, followed by 22 variable-width DIA scans at a resolution of *R* = 35,000 at *m/z* 200, automatically set maximal IT, 2 × 10^5^ automatic gain control target, and a normalized collision energy (NCE) of 27.5. All scans were acquired in the Orbitrap with activated lock mass correction. The cycle time was 3.6 s, ensuring at least 8 scans over the width of a typical liquid chromatography (LC) peak (30 s).

Data analysis was performed using Skyline (version 20.1.0.31) (70) and applying an in-house-built spectral library of peptides identified from previous in-house analysis of H. volcanii. Exported transition peak areas were converted to protein quantities using the MaxLFQ algorithm implemented in the iq R package (71).

Proteins that were quantified in all three biological replicates of either the CRISPRi strain or the wild-type strain but in none of the replicates of the other strain were considered on (only in CRISPRi strain) or off (only in wild-type strain). Proteins that were detected in at least two biological replicates of each strain were included for relative quantification of protein abundance. Data were considered statistically significant when the −log_10_ *P* value was >2. Biological significance was assumed for quantified proteins exhibiting a log_2_ fold change in abundance of >|2| when comparing CRISPRi and wild-type strains. The complete output is shown in Table S4B.

### Western blotting.

The protein expression levels of FtsZ1 and FtsZ2 were assessed by Western blotting using rabbit antisera for FtsZ1 and FtsZ2 (15). FtsZ1 and FtsZ2 rabbit antisera were generated with a synthetic peptide antigen derived from the sequences of the C-terminal regions of FtsZ1 ([C]-Ahx-QAHAEERLEDIDYVE-acid; Cambridge Research Biochemicals, Billingham, UK) and FtsZ2 (NH2-[C]-SDGGRDEVEKNNGLDVIR-COOH; Thermo Fisher Scientific). H. volcanii cell pellets were resuspended in SDS-PAGE sample buffer and then heated (95°C for 5 min) and vortexed. The same amounts of protein were separated by SDS-PAGE, electroblotted (Bio-Rad, Feldkirchen, Germany) onto a nitrocellulose membrane (Protran; Sigma-Aldrich), and probed with rabbit polyclonal primary antiserum (1:1,000 dilution for FtsZ1 and 1:2,000 dilution for FtsZ2), followed by a secondary antibody of donkey anti-rabbit IgG (16284, 1:5,000 dilution; AbCam) conjugated to horseradish peroxidase. Protein bands were detected using an enhanced chemiluminescence detection kit (Thermo Fisher Scientific) and visualized and quantified using an Amersham imager 600 instrument (GE Healthcare). The Western blot signals were quantified using Image J 1.53c software. The relative gray intensities for FtsZ1 and FtsZ2 protein bands were normalized to the lane’s loading control (with Ponceau S staining). All Western blots were performed at least twice with independent biological samples and showed similar results. The Western blot results for one experiment are shown, but quantification was from at least two independent experiments.

### Data availability.

All MS data (raw data, data analysis results, spectral library) have been deposited to the ProteomeXchange Consortium via the PRIDE partner repository (72) with the data set identifier PXD017903.
