# Supplementary figures and images for: CdrS Is a Global Transcriptional Regulator Influencing Cell Division in Haloferax volcanii
Source: mBio. 2021 Jul 13;12(4):e01416-21. doi: 10.1128/mBio.01416-21 (PMC8406309; doi:10.1128/mBio.01416-21)

## Supplementary Figure 1

### Supplementary Figure 1A.

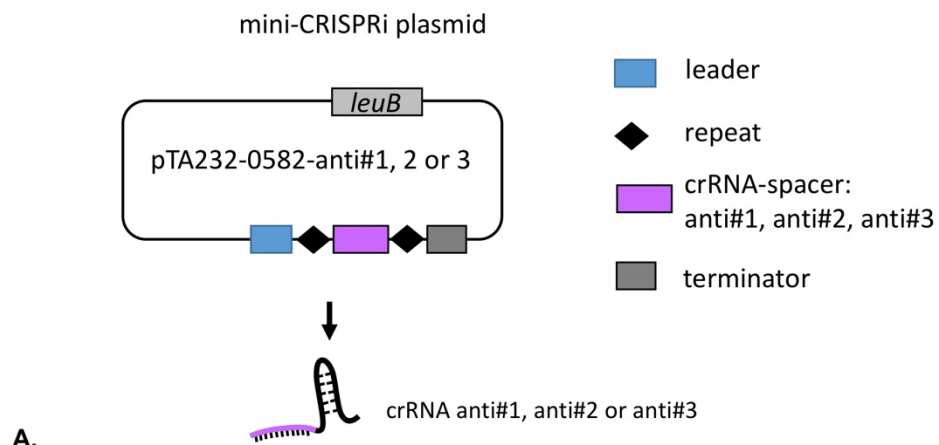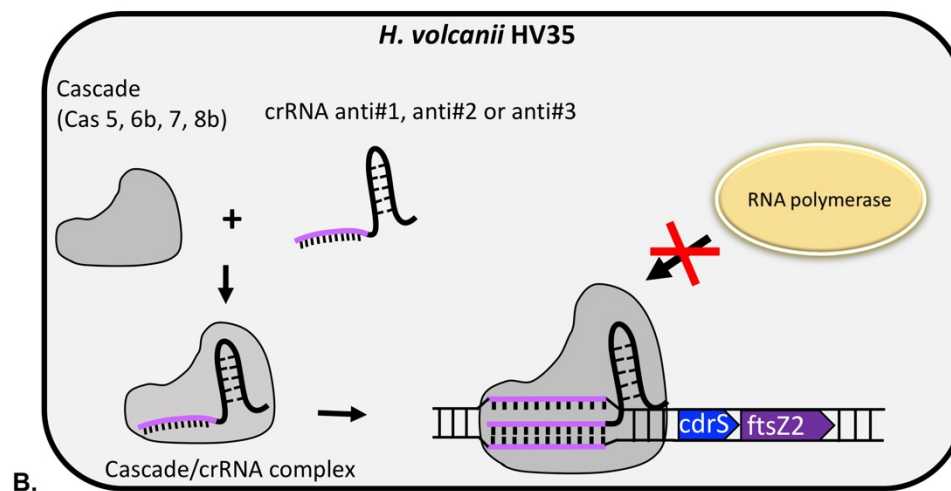

Supplementary Figure 1B.

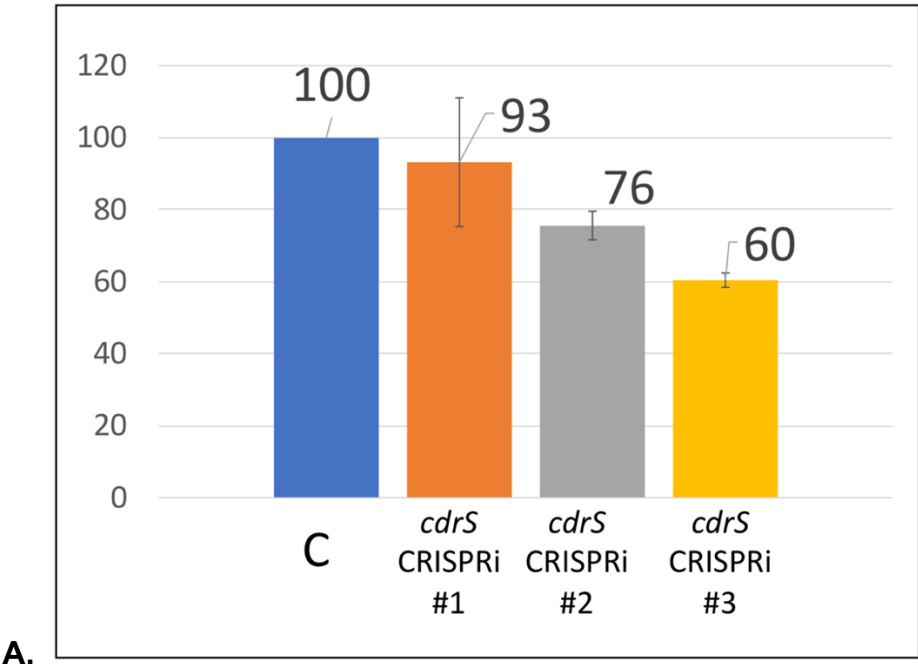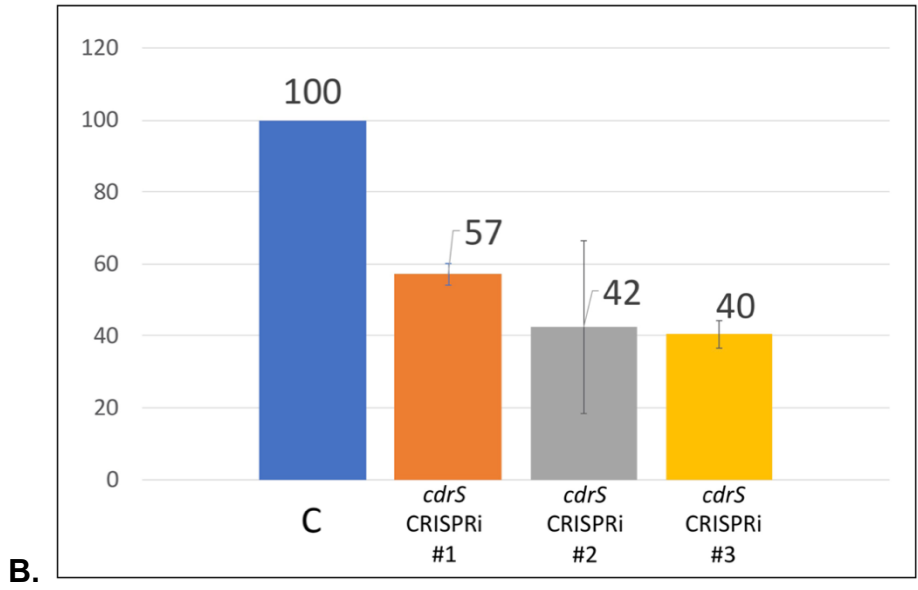

Supplement: FIG S1 [file mbio.01416-21-sf001.pdf]

Supplementary Figure 2.

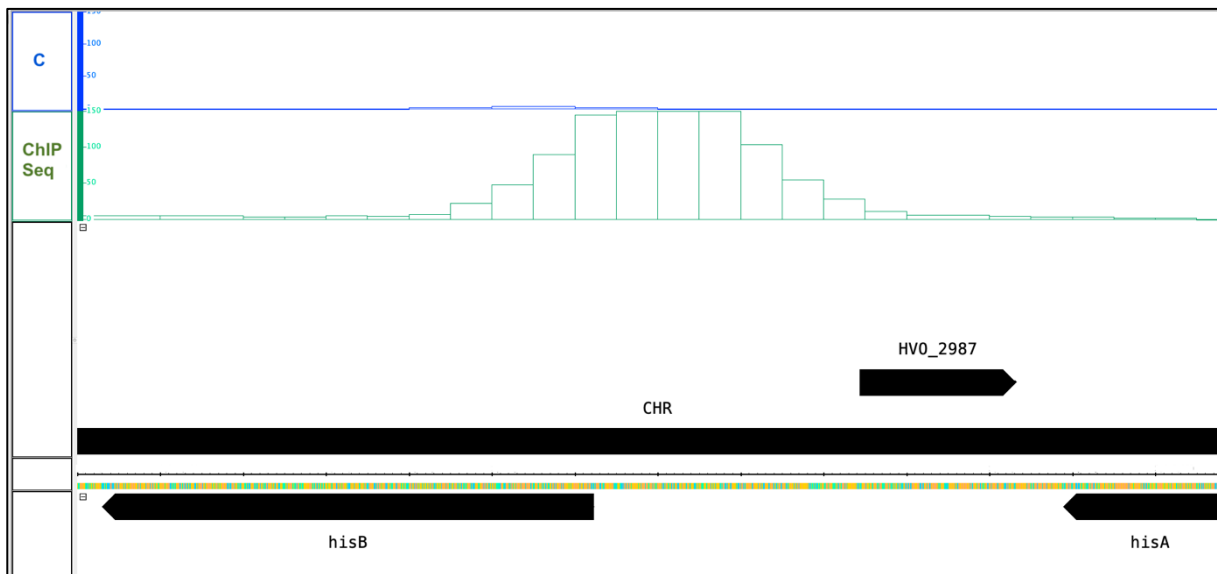

Supplement: FIG S2 [file mbio.01416-21-sf002.pdf]

### Supplementary Figure 3.

#### Supplementary Figure 3A.

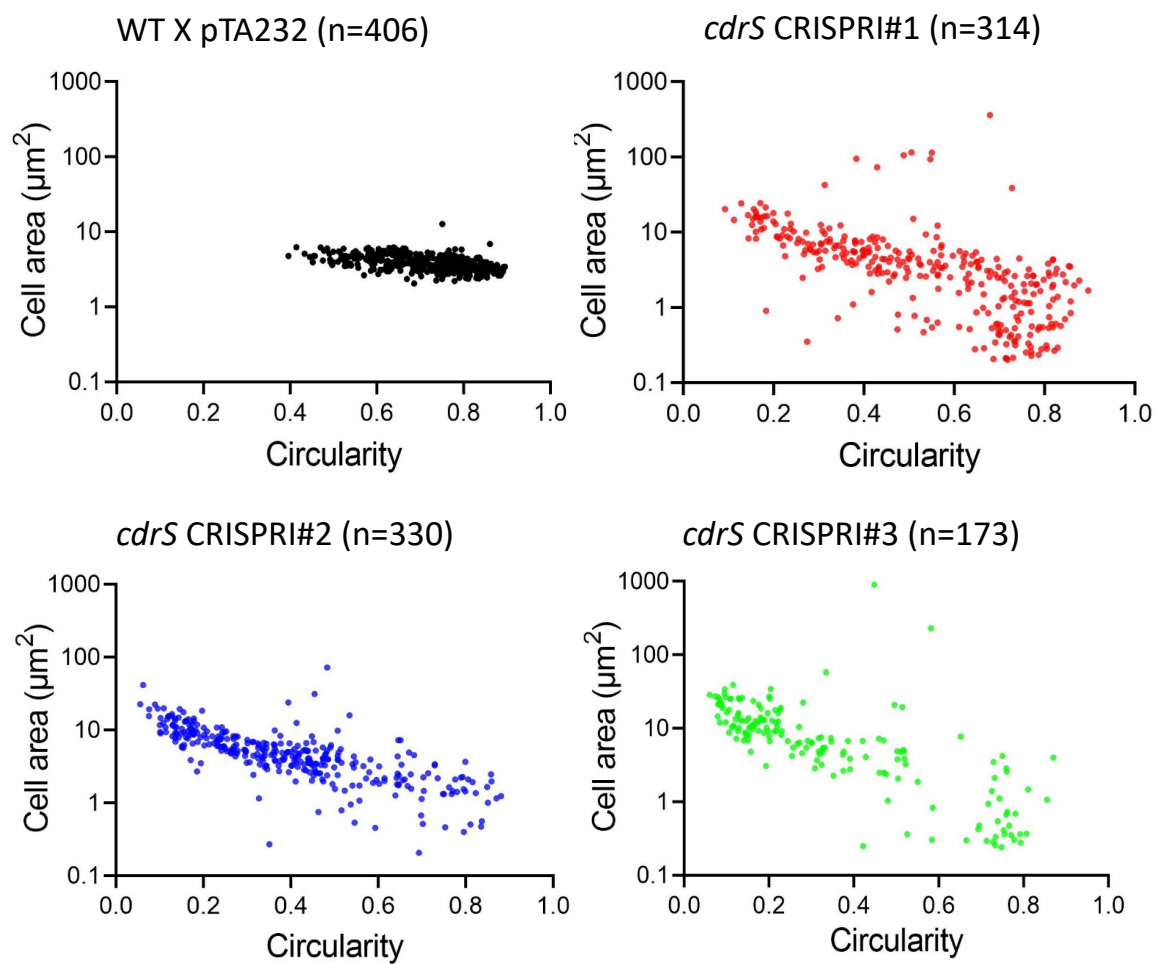

Supplementary Figure 3B.

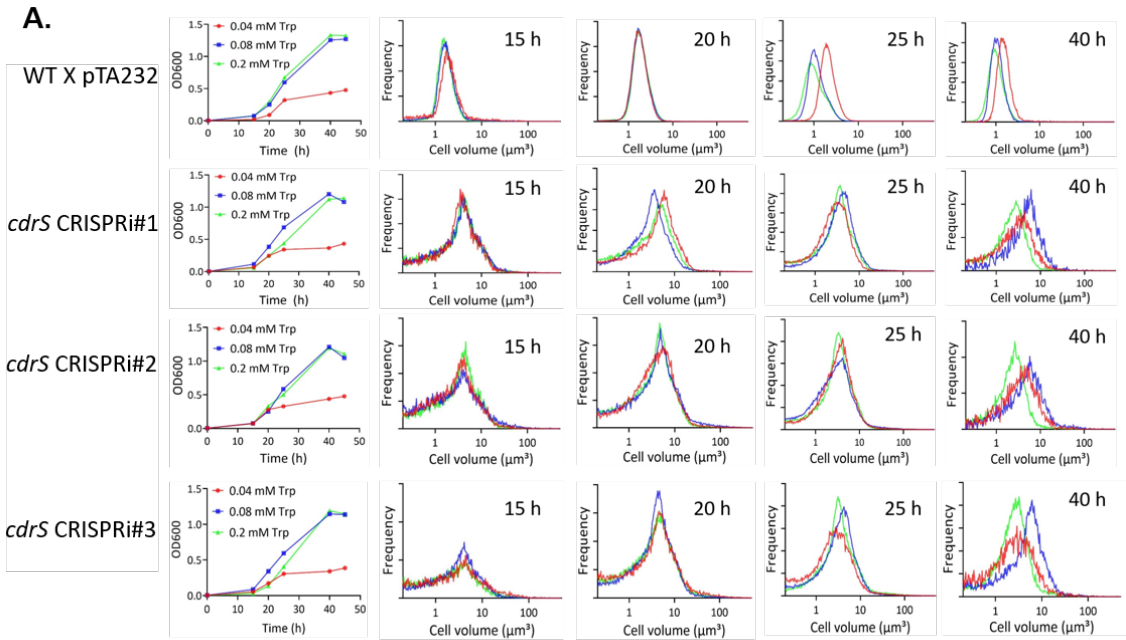

**B.**

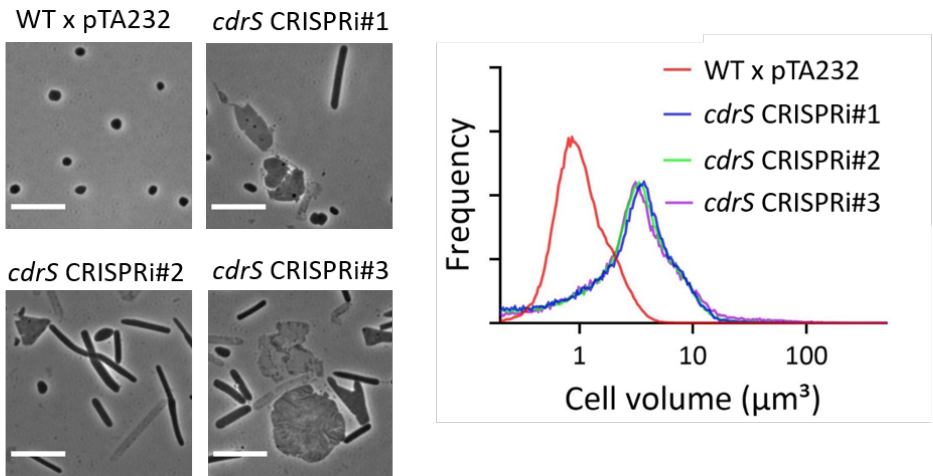

Supplement: FIG S3 [file mbio.01416-21-sf003.pdf]

## Supplementary Figure 4.

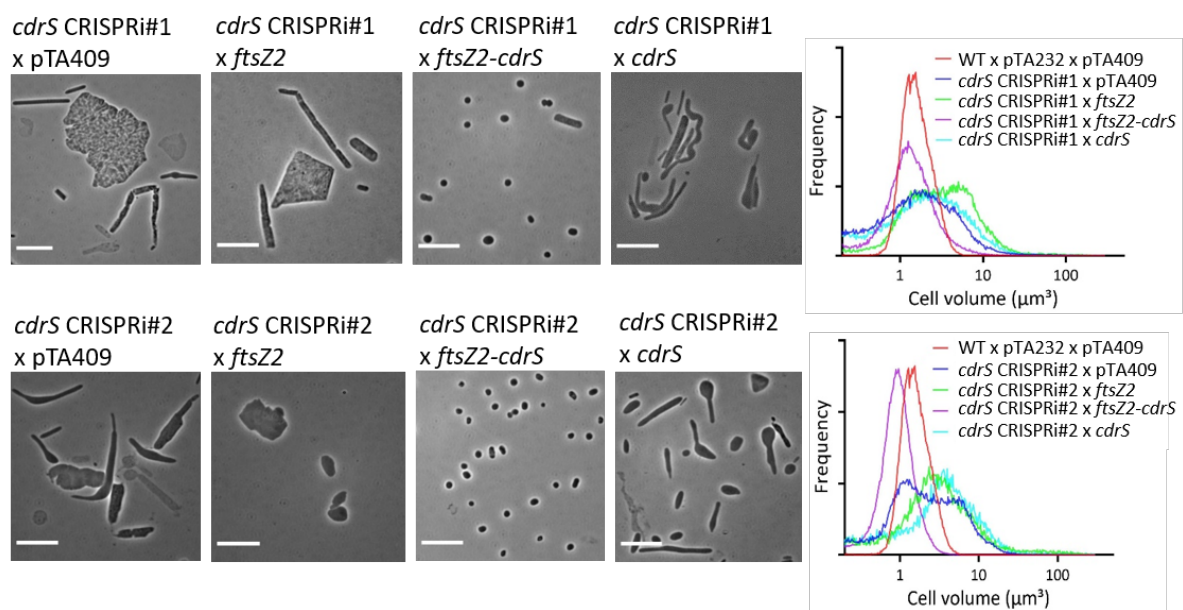

Supplement: FIG S4 [file mbio.01416-21-sf004.pdf]

Supplementary Figure 5.

Supplementary Figure 5A.

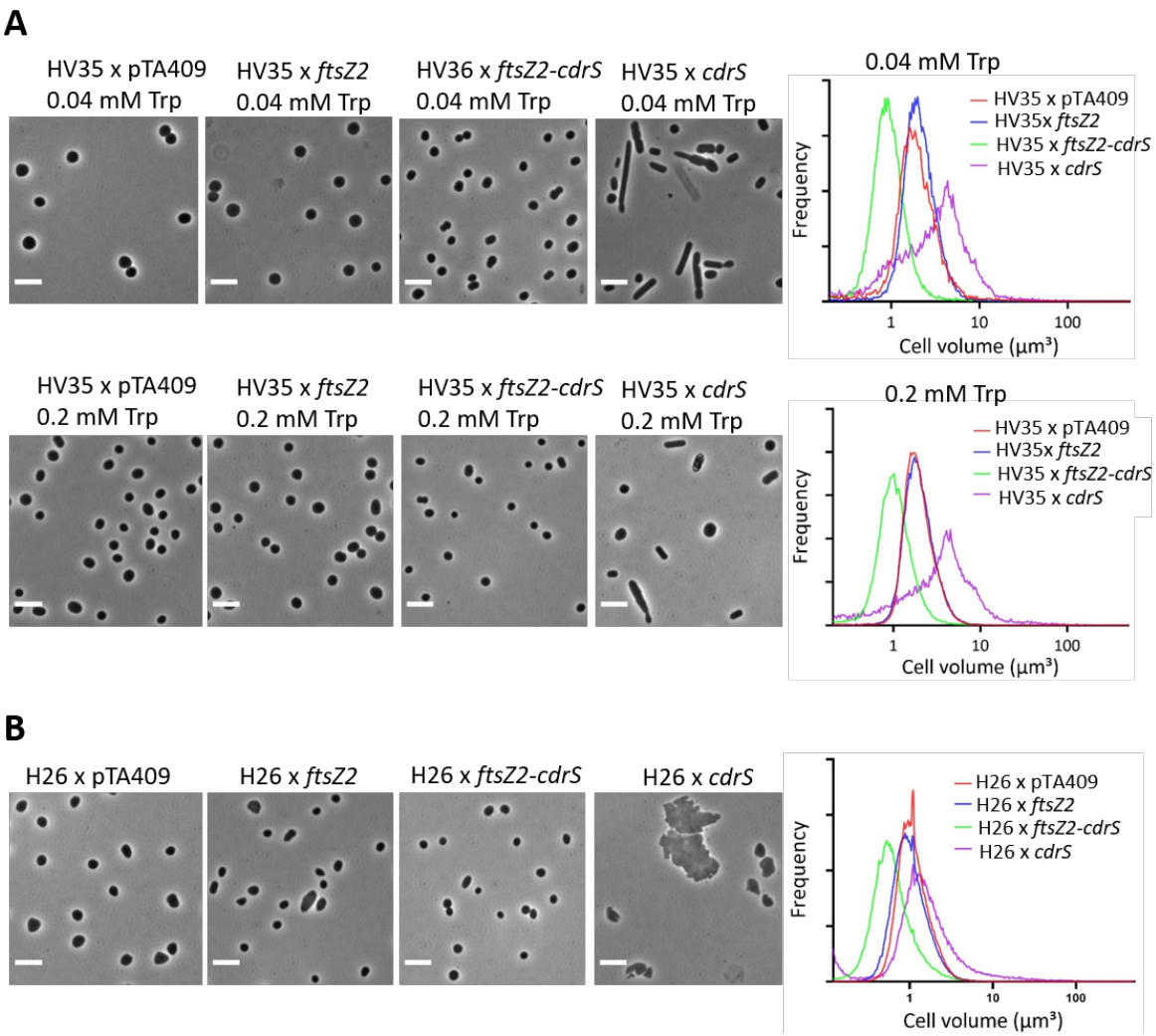

**Supplementary Figure 5B.**

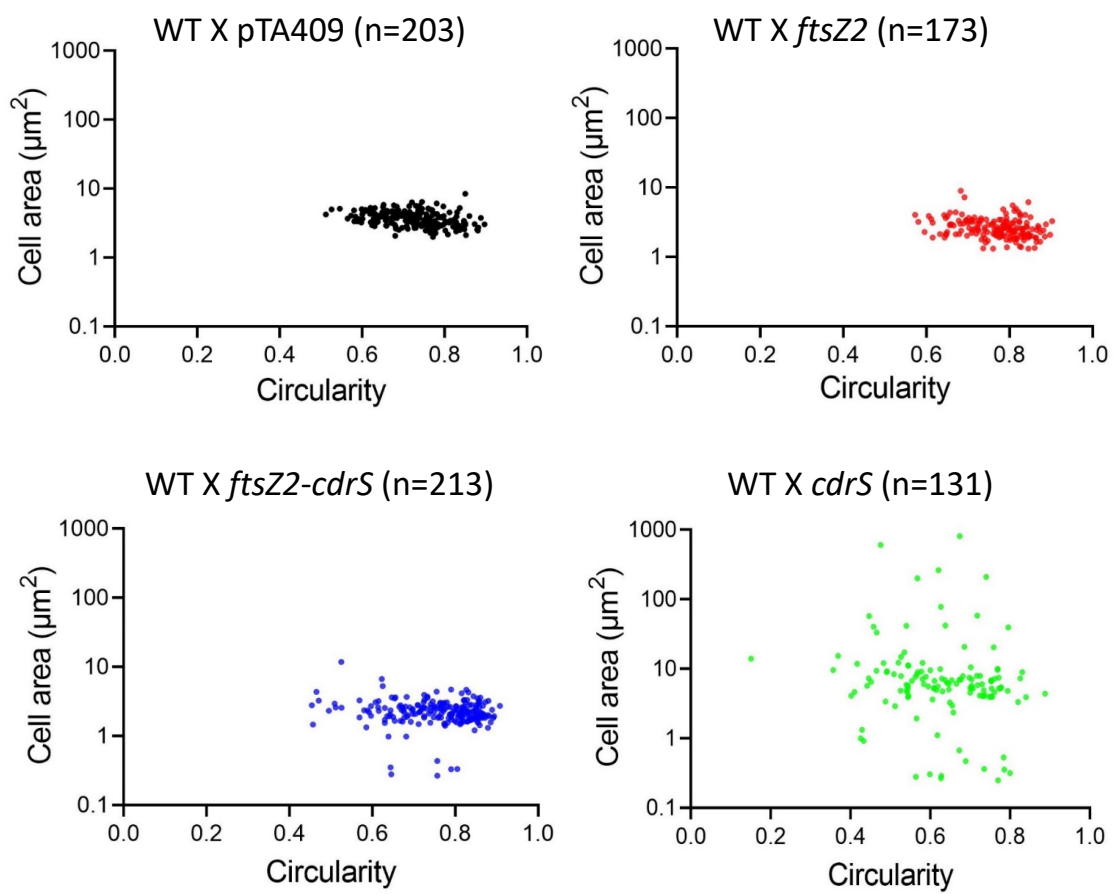

Supplement: FIG S5 [file mbio.01416-21-sf005.pdf]

Supplementary Figure 6.

Supplementary Figure 6A.

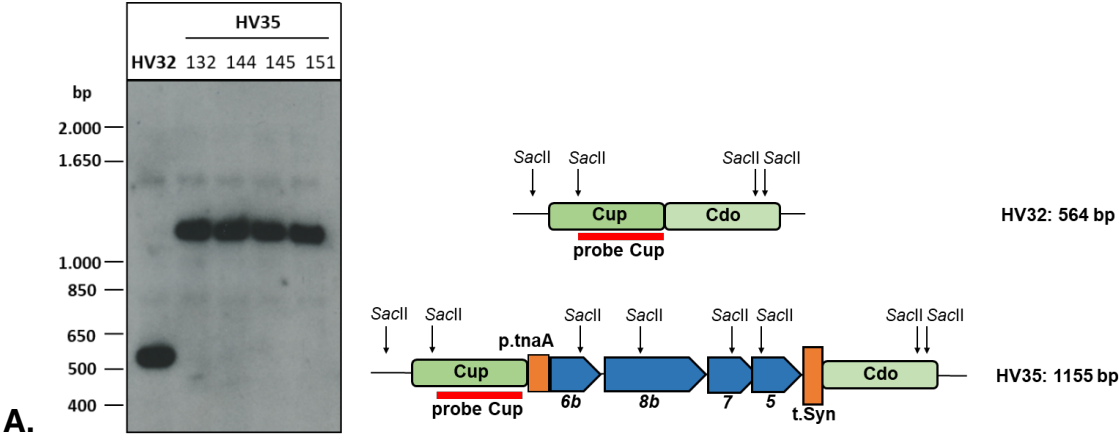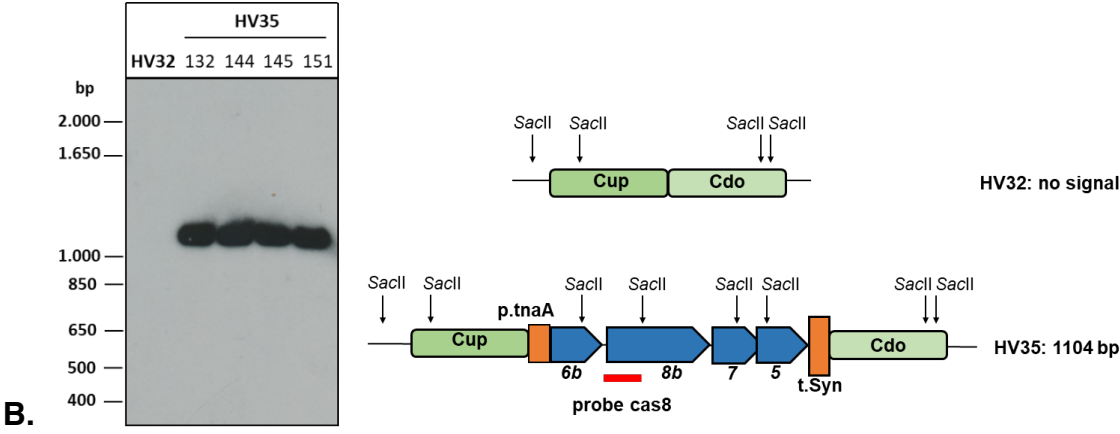

Supplementary Figure 6B.

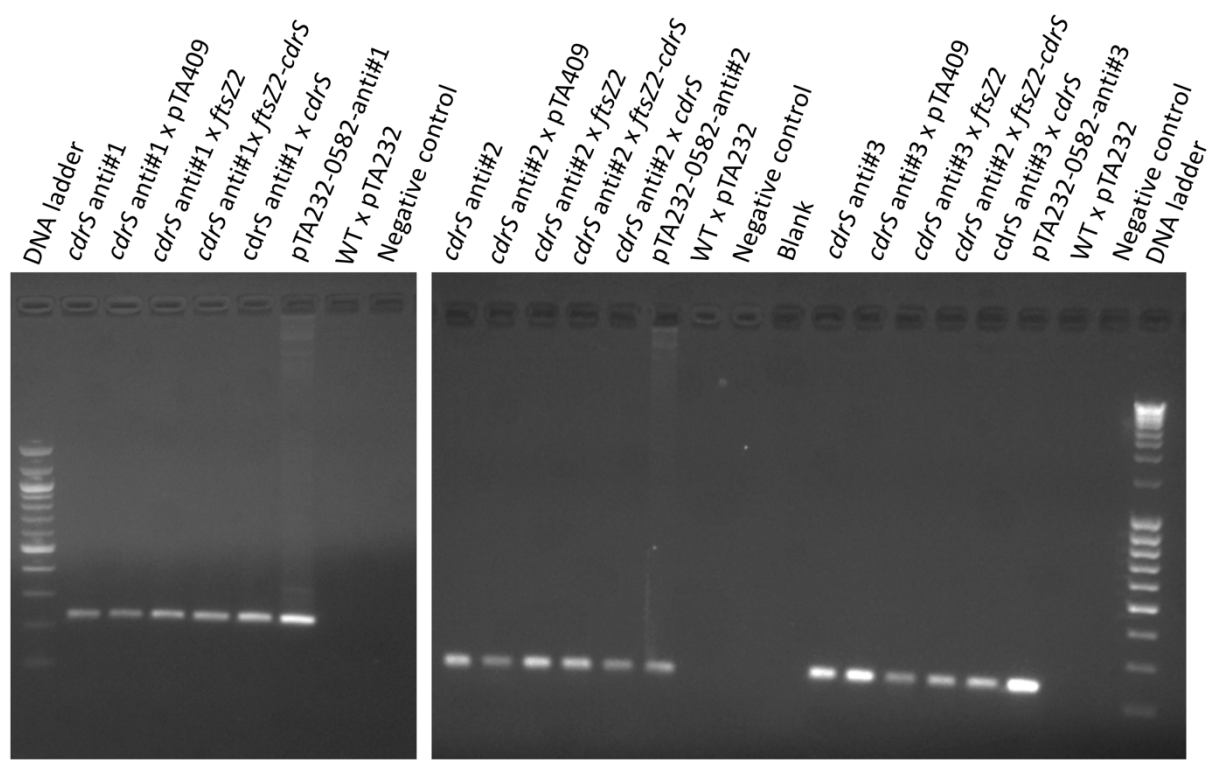

Supplement: FIG S6 [file mbio.01416-21-sf006.pdf]
