# Supplementary material for: CdrS Is a Global Transcriptional Regulator Influencing Cell Division in Haloferax volcanii
Source: mBio. 2021 Jul 13;12(4):e01416-21. doi: 10.1128/mBio.01416-21 (PMC8406309; doi:10.1128/mBio.01416-21)
Supplement: TABLE S1 [file mbio.01416-21-st001.pdf]

Supplementary Table 1A. Genes up- and down regulated in *cdrS-ftsZ2* CRISPRi cells.

I. Upregulated genes

Gene	Annotation	log_2
**Secreted, membrane, and cell surface protein**		
HVO_1003	GufA family transport protein (probable substrate zinc, signal peptide, TMD)	2.0
HVO_B0192*	conserved hypothetical protein (signal peptide, TMD)	2.6
HVO_2323	conserved hypothetical protein (TMD)	1.5
HVO_0814	chaperone (DnaJ domain, TMD)	1.4
HVO_2324	pantothenate permease panF (TMD)	1.2
HVO_B0133	conserved hypothetical protein (TMD)	1.1
HVO_0998	conserved hypothetical protein (signal peptide, TMD)	1.1
HVO_B0132	PQQ repeat protein (signal peptide, TMD)	1.0
HVO_0995	conserved hypothetical protein (TMD)	0.9
HVO_2470	sodium- and chloride-dependent transporter (TMD)	0.8
HVO_0883	conserved hypothetical protein (signal peptide, TMD)	0.7
HVO_1869	conserved hypothetical protein (TMD)	0.7
**Transport**		
HVO_A0339	ABC-type transport system periplasmic substrate-binding protein (probable substrate dipeptides/oligopeptides)	1.0
**Transcription**		
HVO_B0319	IclR family transcription regulator	2.8
HVO_B0193*	ArsR family transcription regulator	2.9
HVO_0576	transcription regulator	0.8
HVO_A0394	conserved hypothetical protein (ArsR-like helix-turn-helix domain)	1.2
**Signal transduction**		
HVO_1358	response regulator	1.3
HVO_1222	CheR-like methyltransferase	1.1
**General metabolism**		
HVO_A0470	dioxygenase	0.9
HVO_2665	HpcH/HpaI aldolase family protein	0.7
**Amino acid metabolism**		
HVO_2646	dihydroxy-acid dehydratase (DHAD)	1.1
**DNA maintenance and repair**		
HVO_1302	DNA-directed DNA polymerase Y polY	1.0
HVO_A0450	universal stress protein 3	0.9
**Genes without known function**		
HVO_A0129	conserved hypothetical protein	1.3
HVO_B0193s2*	RNA of unknown function	3.7
HVO_B0193s*	RNA of unknown function	3.5
HVO_0582s	RNA of unknown function	6.4
HVO_2391s	RNA of unknown function	2.3
HVO_0259s	RNA of unknown function	2.1
HVO_2787s	RNA of unknown function	1.1

HVO_2021	conserved hypothetical protein	1.9
HVO_2392	conserved hypothetical protein	1.8
HVO_0457	Zinc finger protein 330-like protein	1.1
HVO_B0195	Hypothetical protein	1.1

II. Downregulated genes.

Gene	Annotation	$\log_2$
**secreted, membrane, and cell surface protein**		
HVO_0739	conserved hypothetical protein (TMD)	-3.3
HVO_A0152	conserved hypothetical protein (TMD)	-2.2
HVO_A0493	ABC-type transport system permease protein (probable substrate sugar, TMD)	-2.1
HVO_A0173	conserved hypothetical protein (TMD)	-1.9
HVO_2034	putative sugar ABC transporter permease (TMD)	-1.8
HVO_1759	putative iron-III ABC transporter permease (TMD)	-1.7
HVO_2976	carbon starvation protein CstA (TMD)	-1.6
HVO_0607	conserved hypothetical protein (Twin-arginine translocation pathway, signal sequence)	-1.6
HVO_2064	conserved hypothetical protein (signal peptide)	-1.6
HVO_1228	halocyanin (signal peptide)	-1.6
HVO_A0318	conserved hypothetical protein (TMD)	-1.3
HVO_0343	crcB protein-like protein (signal peptide, TMD)	-1.2
HVO_A0165	putative transporter (TMD)	-1.0
HVO_1401	putative sugar ABC transporter periplasmic substrate-binding protein (signal peptide)	-1.0
HVO_1844	conserved hypothetical protein (signal peptide)	-0.9
HVO_B0063	CbtB family protein (TMD) for cobalt transport	-2.4
HVO_B0064	CbtA family protein (signal peptide, TMD) for cobalt transport	-2.8
HVO_B0108	ABC-type transport system permease protein (TMD, MetI-like domain)	-2.3
**Transport**		
HVO_B0217	ABC-type transport system periplasmic substrate-binding protein (probable substrate branched-chain amino acids)	-1.6
HVO_A0494	ABC-type transport system periplasmic substrate-binding protein (probable substrate sugar)	-1.8
**Cell division related**		
HVO_0581	cell division protein FtsZ2	-2.6
HVO_0392	cell division protein SepF	-2.2
HVO_0717	cell division protein FtsZ1	-1.3
HVO_0689	chromosome segregation protein SMC	-1.0
**Transcription**		
HVO_0582	CdrS	-2.5
HVO_0290	ribbon-helix-helix CopG family protein	-1.6
HVO_2110	ArcR family transcription regulator	-1.4
**Cobalamin (vitamin B12) biosynthesis**		
HVO_B0054	sirohydrochlorin cobaltochelatase	-2.1
HVO_B0057	cobalt-factor-III C17-methyltransferase	-2.6

HVO_B0058	cobalt-factor-III C17-methyltransferase	-2.1
HVO_B0059	cobalt-precorrin-5A hydrolase	-2.0
HVO_B0060	cobalt-precorrin-4 C11-methyltransferase	-2.8
HVO_B0061	cobalt-factor-II C20-methyltransferase	-2.7
HVO_B0062	precorrin-8W decarboxylase	-1.6
HVO_A0488	cob(I)alamin adenosyltransferase	-1.6
HVO_0592	adenosylcobinamide amidohydrolase	-1.5
	**General metabolism**	
HVO_A0083	Rieske-type [2Fe-2S] iron-sulfur protein	-2.0
HVO_A0519	monoamine oxidase regulatory protein	-1.8
HVO_A0525	enoyl-CoA hydratase	-1.8
HVO_A0521	phenylacetyl-coenzyme A ligase	-1.7
HVO_0304	electron transfer flavoprotein subunit alpha	-1.6
HVO_2789	putative molybdenum cofactor biosynthesis protein A	-1.4
HVO_0585	putative oxidoreductase	-1.0
HVO_3014	GTP-binding protein Era	-1.0
HVO_1797	mRNA 3´ end processing factor	-0.8
HVO_0212	putative lactoyglutathione lyase	-0.8
HVO_B0238	putative endoribonuclease L-PSP	-1.6
HVO_1983	malate synthase	-1.1
HVO_B0200	malate synthase	-0.9
HVO_B0065	thioredoxin-like superfamily protein	-2.2
	**Amino acid metabolism**	
HVO_0041	ornithine carbamoyltransferase (argF)	-1.4
HVO_0043	acetylornithine aminotransferase (argD)	-1.0
HVO_0046	rimK family protein	-1.0
	**Genes without known function**	
HVO_B0055	conserved hypothetical protein	-2.9
HVO_3013	conserved hypothetical protein	-0.8
HVO_2973	conserved hypothetical protein	-1.8
HVO_B0240	conserved hypothetical protein	-1.6
HVO_2868s	RNA of unknown function	-1.4
HVO_2073s	RNA of unknown function	-1.3
HVO_2351s	RNA of unknown function	-1.2
HVO_1106s	RNA of unknown function	-1.0
HVO_1885s	RNA of unknown function	-2.2

Supplementary Table 1B. Proteins with significant changes in abundance.

		wild-type vs CRISPRi differential abundance (On/Off and log_2 ratio)	
**Gene**	**Annotation**	**Pellet**	**SN**
**Secreted, membrane, and cell surface protein**			
HVO_B0153A	capsule biosynthesis CapC domain protein (signal peptide, TMD)	Off	
HVO_2492	hypothetical protein (TMD)	Off	
HVO_A0629	PAS domain, signal transduction histidine kinase domain (TMD)	Off	
HVO_0739	hypothetical protein (TMD)	-4.96	Off
HVO_2470	sodium- and chloride-dependent transporter SNF		-2.83
HVO_1046	hypothetical protein (TMD)	On	
HVO_2267	hypothetical protein (TMD)	On	
HVO_2027	DoxX domain protein (TMD)		Off
**Transport**			
HVO_1110	ABC-type transport system periplasmic substrate-binding protein (probable substrate cobalamin)	Off	
HVO_A0177	ABC-type transport system ATP-binding protein	On	
HVO_1760	Putative iron-III ABC transporter ATP-binding protein		Off
HVO_2211	TrkA family potassium uptake protein	On	
**Protein synthesis and translation**			
HVO_1148	30S ribosomal protein S15	Off	
HVO_0809	Met-tRNA synthetase	On	
HVO_0870	Pro-tRNA synthetase	On	
HVO_1684	Thr-tRNA synthetase	On	
HVO_0769	TRAM domain-containing protein	On	
**Transcription**			
HVO_B0201	IclR family transcription regulator		Off
HVO_B0320	IclR family transcription regulator		Off
**Carbohydrate metabolism**			
HVO_1172	galE UDP-glucose 4-epimerase	On	
HVO_1494	fructose-1,6-bisphosphate aldolase	On	
HVO_0478	glyceraldehyde-3-phosphate dehydrogenase type II	On	
HVO_1300	triosephosphate isomerase	On	
HVO_2960	Dihydrolipoyllysine-residue acetyltransferase	On	
HVO_B0085	possible polygalacturonase, putative		Off
**Central carbon metabolism**			
HVO_1000	Acetyl-CoA synthetase	On	
**Amino acid metabolism**			
HVO_0044	argB acetylglutamate kinase	On	
HVO_2852	Succinylglutamate desuccinylase		Off
HVO_2992	phosphoribosyl-AMP cyclohydrolas		Off
**Lipid metabolism**			
HVO_2725	Isoprenyl diphosphate synthase (IdsA1)	On	
**General metabolism**			

HVO_0069	arylsulfatase	On	
HVO_0869	glutamate synthase subunit	On	
HVO_0884	Aldehyde reductase	On	
HVO_1874	probable oxidoreductase (aldo-keto reductase family protein)	On	
HVO_0662	ThiN homolog with a predicted N-terminal helix-turn-helix (HTH) DNA binding domain	On	
HVO_1009	Oxidoreductase related to aryl-alcohol dehydrogenases	On	
HVO_2336	pyridoxal 5'-phosphate synthase lyase subunit PdxS)	On	
HVO_2348	GTP cyclohydrolase I	On	
HVO_2650	4-hydroxybenzoate 3-monooxygenase	On	
HVO_2790	ATP-binding protein Mrp	On	
HVO_1697	FAD-dependent oxidoreductase (GlcD/DLD_GlcF/GlpC domain fusion protein)		2.79
HVO_2579	nicotinate-nucleotide pyrophosphorylase (carboxylating)		-2.35
HVO_0510	dihydroneopterin aldolase, archaeal-type, MptD		-2.15
HVO_2127	M20 family amidohydrolase (homolog to indole-3-acetyl-aspartate hydrolase)		Off
HVO_2848	probable PrkA-type serine/threonine protein kinase, PrkA1		Off
**DNA maintenance and repair**			
HVO_2452	ribonucleoside-diphosphate reductase, adenosylcobalamin-dependent	On	
HVO_0203	Replication factor C small subunit	On	
HVO_0104	DNA repair and recombination protein RadA	3.84	
HVO_2911	deoxyribodipyrimidine photo-lyase, phr1		Off
**Cell division-related**			
HVO_0392	SepF		-3.32
HVO_0581	FtsZ2		-3.19
**Proteins without known function**			
HVO_2519	hypothetical protein	On	
HVO_0400	hypothetical protein	On	
HVO_1535	hypothetical protein		Off
HVO_0508	hypothetical protein	On	
HVO_1377	uncharacterised protein family UPF0145	On	
HVO_0577	uncharacterised DUF2028 domain		-4.55

14
