# Supplementary material for: CdrS Is a Global Transcriptional Regulator Influencing Cell Division in Haloferax volcanii
Source: mBio. 2021 Jul 13;12(4):e01416-21. doi: 10.1128/mBio.01416-21 (PMC8406309; doi:10.1128/mBio.01416-21)
Supplement: TABLE S2 [file mbio.01416-21-st002.pdf]

Supplementary Table 2.

	WT X pTA232 (n=406)	*cdrS* CRISPRi#1 (n=314)	*cdrS* CRISPRi#2 (n=330)	*cdrS* CRISPRi#3 (n=173)	WT x *cdrS* (n=131)
Filaments	0 %	20 %	30 %	52.6 %	2.3 %
Giant cells	0 %	5.7 %	3 %	4.6 %	45 %
Wild-type like	100 %	43 %	52.4 %	24.9%	40.5 %
Cellular debris	0 %	31.2 %	14.5 %	18 %	12.2 %
