# Supplementary material for: CdrS Is a Global Transcriptional Regulator Influencing Cell Division in Haloferax volcanii
Source: mBio. 2021 Jul 13;12(4):e01416-21. doi: 10.1128/mBio.01416-21 (PMC8406309; doi:10.1128/mBio.01416-21)
Supplement: TABLE S3 [file mbio.01416-21-st003.pdf]

Supplementary Table 3.

Supplementary Table 3A.

**Strains**	**Genotype**	**Reference**
H26	ΔpHV2, Δ*pyrE2*	(Allers, Ngo, Mevarech, & Lloyd, 2004)
HV30	ΔpHV2, Δ*pyr*E2, Δ*trp*A, Δ*leu*B, Δ*cas*6, Δ*cas*3, Δ*bga*H	(Stachler & Marchfelder, 2016)
HV32	ΔpHV2, Δ*pyrE2*, Δ*trpA*, Δ*leuB, ΔI*-B, ΔHVO_2.385.045–2.386.660, ΔHVO_pHV4: 204.834-218.566	(Stachler & Marchfelder, 2016)
HV35	ΔpHV2, Δ*pyrE2*; Δ*leuB*; Δ*trpA*; ΔHVO_2,385,045–2,386,660::p.tna,*cas6*,*cas8*, *cas7*,*cas5*, t.syn; ΔHVO_pHV4:204,834-218,566	this study
DH5α	F- 80*lac*ZΔM15 Δ(*lac*ZYA-*arg*F) U169 *rec*A1 *end*A1 *hsd*R17 (rk-, mk+) *gal*- *pho*A *sup*E44λ- *thi*-1 *gyr*A96 *rel*A1	Invitrogen (Thermo Fisher Scientific, Waltham, USA)
GM121	F- *dam-3 dcm-6 ara-14 fhuA31 galK2 galT22 hdsR3 lacY1 leu-6 thi-1 thr-1 tsx-78*	(Allers, Barak, Liddell, Wardell, & Mevarech, 2010)

Supplementary Table 3B.

plasmids	relevant properties	Reference/ source
pBlueScriptII	*E. coli* plasmid	Stratagene
pBlue-HVO_0582	*E. coli* plasmid with HVO_0582 *gene*	this study
pTA232	Shuttle vector with *leu*B marker and pHV2 replication origin	(Allers et al., 2004)
pTA231	Shuttle vector with *trpA* marker, pHV2 replication origin	(Allers et al., 2004)
pTA409	Shuttle vector with *pyrE*2 marker and pHV1 replication origin	(Delmas, Shunburne, Ngo, & Allers, 2009)
pTA131-up-HVO_0582-do	ColE1 ori, f1 ori*, lac*Z*,* AmpR*, pyr*E2, gene HVO_0582 with 500 bp flanking upstream and downstream regions	this study
pTA131-up-ΔHVO_0582-do	ColE1 ori, f1 ori*, lac*Z*,* AmpR*, pyr*E2, 500 bp flanking regions upstream and downstream of HVO_0582	this study
pTA131-Cup-p.ntaA-cas6b8b75-t.Syn-Cdo	ColE1 ori, f1 ori, *lacZ*, AmpR, *pyrE*2, Cup-p.ntaA*cas6bcas8bcas7cas5*-t.syn-Cdo	this study
pMA-telecrRNA	*E. coli* plasmid with promoter, crRNA against spacer C1, flanked by t-Elements, terminator	(Maier, Dyall-Smith, & Marchfelder, 2015)
pTA927-ptnaA-NFlag	Shuttle vector with *pyrE2* marker, pHV2 replication origin, a tryptophan inducible promoter and a 3xFLAG tag cDNA	(Fischer et al., 2010)
pMK-RQ-0582anti#1	*E. coli* plasmid containing the promoter, spacer sequence flanked by Haloferax repeats and terminator, expressing a crRNA#1 against the HVO_0582 RNA gene	GeneArt (Thermo Fisher Scientific)
pMK-RQ-0582anti#2	*E. coli* plasmid containing the promoter, spacer sequence flanked by Haloferax repeats and terminator, expressing a crRNA#2 against the HVO_0582 RNA gene	GeneArt (Thermo Fisher Scientific)
pMK-RQ-0582anti#3	*E. coli* plasmid containing the promoter, spacer sequence flanked by Haloferax repeats and terminator, expressing a crRNA#3 against the HVO_0582 RNA gene	GeneArt (Thermo Fisher Scientific)
pTA232-0582anti#1	Plasmid containing the promoter, spacer sequence flanked by *Haloferax* repeats and terminator, expressing a crRNA#1 against the template strand of the HVO_0582 gene	this study
pTA232-0582anti#2	Plasmid containing the promoter, spacer sequence flanked by *Haloferax* repeats and	this study

	terminator, expressing a crRNA#2 against the template strand of the HVO_0582 gene	
pTA232-0582anti#3	Plasmid containing the promoter, spacer sequence flanked by *Haloferax* repeats and terminator, expressing a crRNA#3 against the template strand of the HVO_0582 gene	this study
pMA-tele-anti#1	*E. coli* plasmid with promoter, crRNA anti#1 against HVO_0582 , flanked by t-elements, terminator	this study
pMA-tele-anti#2	*E. coli* plasmid with promoter, crRNA anti#2 against HVO_0582 , flanked by t-elements, terminator	this study
pMA-tele-anti#3	*E. coli* plasmid with promoter, crRNA anti#3 against HVO_0582 , flanked by t-elements, terminator	this study
pTA232-tele-anti#1	pTA232 plasmid with promotor, crRNA anti#1 against HVO_0582, flanked by t-elements, terminator	this study
pTA232-tele-anti#2	pTA232 plasmid with promotor, crRNA anti#2 against HVO_0582, flanked by t-elements, terminator	this study
pTA232-tele-anti#3	pTA232 plasmid with promotor, crRNA anti#3 against HVO_0582, flanked by t-elements, terminator	this study
pTA927-ptnaA-HVO_0582NFlag	Plasmid pTA927 with tryptophan inducible promoter, HVO_0582 fused to an N-terminal 3xFlag tag	this study
pTA231-pfdx-HVO_0582NFlag	Plasmid pTA231 with HVO_0582 fused with an N-terminal Tag under the control of the pfdx promoter	this study
pTA409-pfdx-HVO_0582-nat.t	Plasmid expressing the gene HVO_0582 under the control of the pfdx promotor and the natural terminator	this study
pTA409-pfdx-HVO_0581-nat.t	Plasmid expressing the gene HVO_0581 under the control of the pfdx promotor and the natural terminator	this study
pTA409-pfdx-HVO_0582-HVO_0582-nat.t.	Plasmid expressing the operon HVO_0582-HVO_0581 under the control of the pfdx promotor and the natural terminator	this study

Supplementary Table 3C.

primer	5’->3’ sequence
RS	CACAGGAAACAGCTATGACC
US	GTAACGCCAGGGTTTTCCC
P1upfw	GCGTCGGCTCGATTCCACTCACCAACG
P2dorev	CCTCGACGCCGTCGAGAGACTCGAATC
anti#2 fw	(Phos)GCGTAAGACGGTTGTCTTGTTTCAGACGAACCCTTGTGG
anti#2 rv	(Phos)GATAATCACCACAGAGGGGCTTCAACTACCGATCAACG
anit#3 fw	(Phos)ACCCGACGGCGGCCCGTAGTTTCAGACGAACCCTTGTGG
anti#3 rv	(Phos)AGCTGTAAGACAACCGTCGCTTCAACTACCGATCAACG
5’-HVO_0582-*Hind*III	AAGCTTATGGAGCGTGTGACACTACGAATTCC
3’-HVO_0582-*Xba*I	TATATCTAGATTACACCTTTGCCCAGCCGCG
5‘-HVO_0582-*Nde*I	TATTACATATGGAGCGTGTGACACTACGAATTCC
3'-HVO_0582-*Hind*III	TATATAAAGCTTTTACACCTTTGCCCAGCC
5‘-HVO_0581-*Nde*I	TATATACATATGATGCAGGATATCGTTCGC
3'-HVO_0581-nat.t.-*Apa*I	TATATAGGGCCCCTCGCGGTCGAAGAAATC
5‘-*Hind*III-nat.t	TATATAAAGCTTCGCCCTGTCCGACCCGCG
5-HindIII-Cas8	TATTATAAGCTTACAGGTCCAGATATCGACGACTTCG
8R126A#2	CCACGAACGAACGGCTCCGAGAATCGTGCTGGC
CdelupKpnI	TATAGGTACCCGCTCGTCGGTGAGTCGCTCACCGACTTCCG
CdelupiEcoRV	TATAGATATCCGAGGCGGAGCGTCGAGAGCGCTAGTC
3'-HVO_B0192-NB-rev	GCGAGCGAGACGATAAGCCAGTCG
5'-HVO_B0193-NB-fw	CAACCATTACAGCGTCTACACCGC
5'-HVO_0739-NB-fw	CGGGGGGTTCCTCCTCGGCGTGC
3'-HVO_0739-NB-rev	GAAGAGCGCGACGCCGAGGACGGC
anti#1 fw	(Phos)GATTATCGCGTAAGACGGGTTTCAGACGAACCCTTGTGG
anti#2 rev	(Phos)ACCACAGAGGGATAGAATGCTTCAACTACCGATCAACG
HVO_0582-UP	TCGAACGGCGAAT CTCGCGTAAACGC
HVO_0582-DO	CCTCGGCCTTGAC CGTGCGGGCGC
iPCR_HVO_0582-Do	[P]CAGCAAGCGCG GCTGGGCAAAG
iPCR_HVO_0582-UP	[P]GTTTACATTCCC CCGGTAAGACGG
